# Direct and Indirect Linkages Between Trace Element Status and Health Indicators - a Multi-tissue Case-Study of Two Deer Species in Denmark

**DOI:** 10.1007/s12011-023-03926-3

**Published:** 2023-11-02

**Authors:** Floris M. van Beest, Niels M. Schmidt, Monica Lønborg Frederiksen, Anne K.H. Krogh, Heidi H. Petersen, Sophia V. Hansson

**Affiliations:** 1 Department of Ecoscience, Aarhus University, Frederiksborgvej 399, DK-4000 Roskilde, Denmark; 2 Vet Consult, Skovvej 58, 2920 Charlottenlund, Denmark; 3 Department of Veterinary Clinical Sciences, University of Copenhagen, Dyrlægevej 16, DK-1870 Frederiksberg, Denmark; 4 Center for Diagnostics, Technical University of Denmark, Kemitorvet, DK-2800 Kgs. Lyngby, Denmark; 5 Laboratoire Ecologie Fonctionnelle et Environnement (UMR- 5245), CNRS, Université de Toulouse, Ave. de l’Agrobiopole, 31326 Castanet Tolosan, France

**Keywords:** Body condition score, Ecogeochemistry, Serum biochemistry, Structural equation models, Ungulates, Wildlife health

## Abstract

Measuring trace element concentrations in tissue can be a valuable approach to monitor animal health status. Temporal variation in the absorption, transport, and storage of elements between different tissues can, however, complicate the assessment of element-health relationships. Here, we measured concentrations of selected essential (copper (Cu), zinc (Zn), selenium (Se)) and non-essential (arsenic (As), cadmium (Cd), lead (Pb)) trace elements within blood, liver, kidney, and hair of fallow deer (*Dama dama*; *N*=20) and red deer (*Cervus elaphus*; *N*=21). Using multivariate regression and structural equation models, we estimated direct and indirect linkages between tissue-specific trace element profiles and long- (body condition) and short-term (serum protein biomarkers for acute inflammation, infection, and malnutrition) health indicators. Trace element concentrations varied markedly and were weakly correlated among tissues, with the exception of Se. After accounting for sex- and site-differences in trace element concentrations, body condition of red deer was directly, and positively, associated to trace element status in liver and hair, but not in kidney. For both deer species, trace element status in blood was directly linked to serum protein status with an indirect positive association to deer body condition. For fallow deer, no direct association between trace element status and body condition was detected in any of the tissues, possibly because of elemental homeostasis, and because all individuals were in good clinical health. This study shows that hair can serve as an effective, non-invasive, biomarker in deer health assessments, yet, to fully uncover trace element-health relationships a variety of sample matrices is preferred.

**Supplementary Information:**

The online version contains supplementary material available at 10.1007/s12011-023-03926-3.

## Introduction

The fields of ecogeochemistry and biological stoichiometry have made great progress in understanding how the uptake and regulation of different chemical elements can influence the fitness and overall health of organisms [1–3]. Specifically, trace elements such as copper (Cu), zinc (Zn), and selenium (Se) are considered essential to the growth, reproduction, and survival of many plant and animal species [4, 5]. Adverse health effects may arise, however, if homeostasis is disrupted, i.e., when reserves of essential elements are outside required physiological ranges [6, 7]. In addition, some trace elements such as arsenic, (As), cadmium (Cd), and lead (Pb), are considered non-essential as they hold no physiological function and can suppress performance of organisms already at low concentrations, often with long-lasting adverse health effects [8–10].

In many parts of the world, deer species constitute a culturally and economically valuable resource, providing meat and income through hunting and tourism [11]. Monitoring the health status of deer is, thus, a critical component of successful conservation and management of free-ranging populations and fenced herds alike [12–14], with possible implications for human health [15]. Assessments of trace element profiles in free-ranging species have conventionally been done on liver and kidney samples from culled animals, as these storage organs are expected to most accurately reflect elemental status and health of individuals [16, 17]. In live animals, however, whole blood is the most commonly used sample tissue to assess element status [18], though the turnover rate of element concentrations in blood is high and correlations with other tissues are often poor in free-ranging and domestic ungulates [19, 20]. The use of wool or hair as an alternative, non-invasive, indicator of trace element status in large herbivores is rapidly gaining traction [17, 21–24] with documented linkages to individual health and population performance [25–27].

Monitoring the elemental status of animals using tissue samples and estimating linkages to condition or health can, however, be challenging due to temporal variation in the absorption, transport, and storage of trace elements across different tissues [28]. Moreover, the health status of an organism is multidimensional and often difficult to measure fully, especially in free-ranging, wild animals [29]. Oftentimes, the health status of wildlife is approximated by measuring physiological and morphological parameters that vary over seasonal timescales [29]. In some cases, blood samples are taken to measure serum protein levels that are useful in the diagnosis of acute inflammation or infection [30, 31]. Yet, an understanding of how such proxies relate to the elemental status measured in different tissues and long- and short-term health impacts remains unclear, though linkages are slowly being uncovered [32–34].

The main aim of this study was to conceptualize and estimate potential direct and indirect pathways by which trace element status within different tissues can influence the multidimensionality of wildlife health. Using fallow deer (*Dama dama*) and red deer (*Cervus elaphus*) as model species, we first quantified variation and cross-correlation of the essential (Cu, Zn, Se) and non-essential (As, Cd, Pb) trace element concentrations within and among blood, liver, kidney, and hair samples. As traditionally done in livestock and wildlife health assessments, we used multivariate regression to estimate how the various essential and non-essential trace elements were associated to a single, long-term (weeks to months) health indicator (i.e., body condition). To assess potential direct and indirect linkages between trace element status (i.e., the essential and non-essential elements combined) in the various tissues and deer health, we constructed a flexible analytical framework that jointly considered body condition and several short-term (days to weeks) health indicators derived from serum protein analyses. We expected trace element status in storage tissue (liver, kidney) to be directly associated to the long-term health indicator body condition. In contrast, we expected trace element status in transport tissue (blood) to be directly associated to serum protein status, with an indirect linkage to body condition. Finally, and since trace element concentrations tend to accumulate in hair, we expected potential health pathways derived from elemental status in this tissue type to be comparable to those detected with storage tissues liver and kidney.

## Materials and Methods

### Study Animals and Sites

**Fig. 1:**
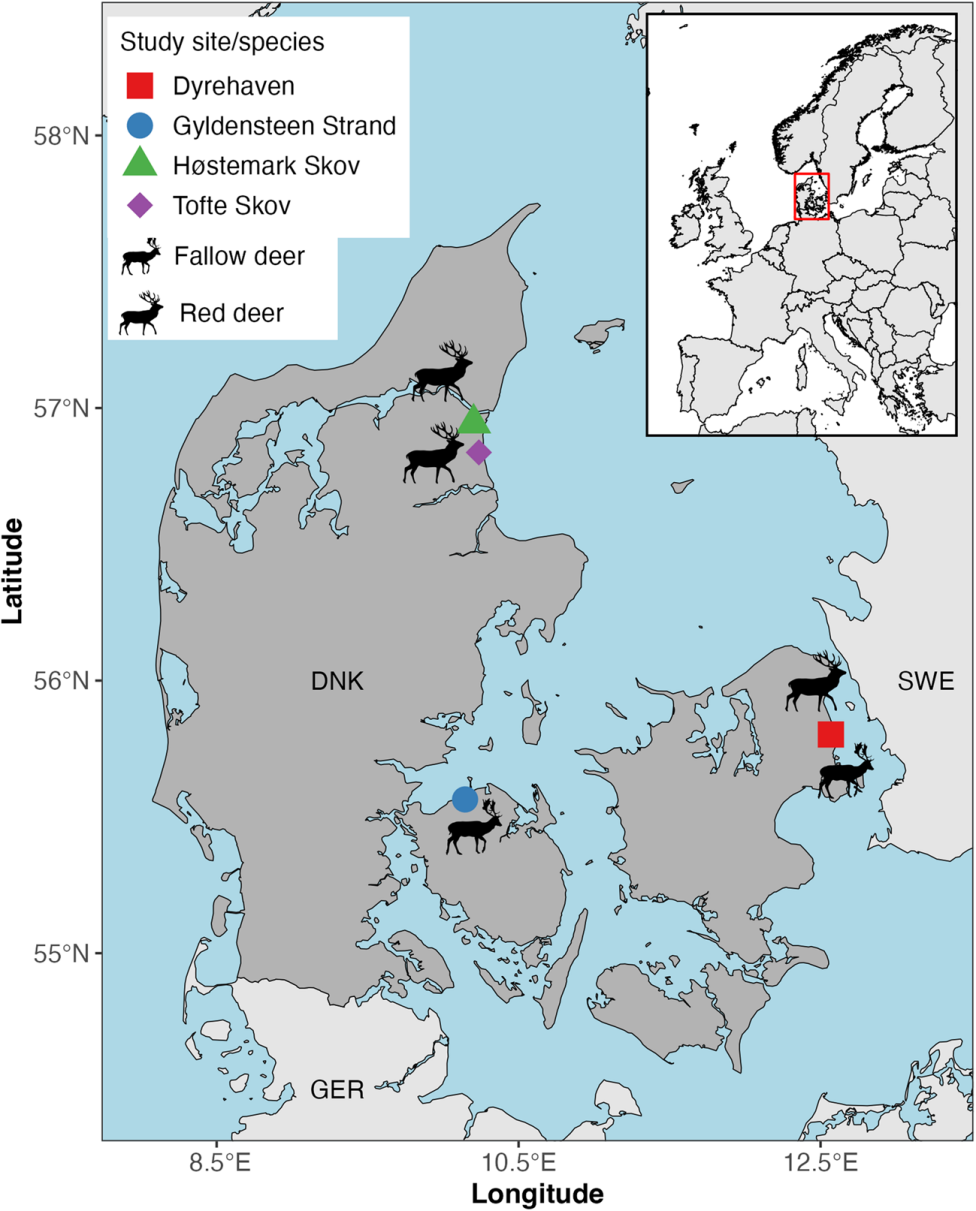
Map of Denmark (DNK) and its position in Europe (see red square in inset) showing the locations of the four study sites (Dyrehaven — red square; Gyldensteen Strand — blue circle, Høstemark Skov— green triangle; Tofte Skov– purple diamond) where fallow deer (*Dama dama*; *N*=20) and/or red deer (*Cervus elaphus*; *N*=21) were sampled. Marine areas and fjords are shown in lightblue

This study is based on tissue samples collected from culled fallow deer and red deer at four different locations in Denmark (Fig. 1). No animals were culled for the purpose of this study and all deer were shot by certified local hunters (using lead-free rifle ammunition) as part of the annual deer management and regulation programs. All deer were sampled post-mortem and culled between November 8th and December 20th in 2021, which is within the official hunting season in Denmark. Sex and age-class were recorded for each individual deer. Age-classes (juvenile, sub-adult, adult) were determined based on tooth wear, body-, and/or antler size characteristics sensu: [35, 36].

The climate in Denmark (and the four study sites) is temperate with a mean annual temperature of ca. 8.3°C and a mean annual precipitation of ca. 750 mm. Three of the study sites were fenced estates (Dyrehaven, Tofte Skov, and Høstemark Skov), while Gyldensteen Strand was unfenced.

Dyrehaven estate (1000 ha) has been fenced for 350 years and has clay-rich soils. The area is managed as a natural forest containing large oak (*Quercus* spp.), beech (*Fagus* spp.), and chestnut (*Castanea* spp.) trees interspersed by grasslands. The estate has not been cultivated since 1700 and deer are supplementary fed from November to April with wholecrop silage bales containing oats, grass, and sugar beet. Dyrehaven had an estimated spring (before calving) density of 23 red deer per 100 ha and 115 fallow deer per 100 ha. In Dyrehaven, deer were shot using tin-based rifle ammunition.

Tofte Skov (4000 ha) and Høstemark Skov (570 ha) have been fenced for nearly 120 years and both estates are characterized by raised peat bog with sandy, acidic soils, which is ideal for purple-moor grass (*Molinia caerulea*) and bushgrass (*Calamagrostis epigejos*). The forests are dominated by beech (*Fagus* spp.), birch (*Betula* spp.), spruce (*Picea* spp.), and alder (*Alnus* spp.). At both estates mineral licks are provided to deer year-round and at Høstemark Skov deer are also supplementary fed during winter with wholecrop silage bales containing oats, grass, and peas. Tofte Skov had an estimated spring density of 10 red deer per 100 ha and Høstemark Skov had an estimated spring density of 25 red deer per 100 ha. No fallow deer were present in Tofte- and Høstemark Skov and red deer were shot using copper-based rifle ammunition at both sites.

At Gyldensteen Strand (~600 ha), fallow deer are free-roaming and the most abundant deer species with only few red deer present in the area (none were included in this study). Fallow deer from Gyldensteen Strand were shot with both copper and tin-based rifle ammunition. The area was shallow intertidal habitat until 1871 when it was reclaimed for agricultural use. In 2014, 211 ha of the area were permanently flooded with seawater as part of a nature restoration program. The area has sandy soils and is characterized by reed beds. The surrounding area is used for agriculture, primarily to produce barley (*Hordeum vulgare*), maize (*Zea mays*), and rapeseed (*Brassica napus*).

### Deer Sampling and Sample Treatment

The body condition of individual deer was assessed by scoring the amount of muscle and subcutaneous fat at multiple locations on the body (i.e., body condition scores (BCS)) following the method proposed by Audige et al. [37]. Specifically, BCSs were estimated by visually inspecting and palpating the chest, ribs, spine, hips, rump, and the base of the tail bone to assess muscle mass and fat deposits and scoring the overall condition between 1, representing “very poor condition” (cachexia or emaciated), and 5, representing “very good condition” (fat) using half-unit increments. The BCS of all deer were assessed by the same professional wildlife veterinarian to limit observer bias and maintain consistency [38].

Blood (60 ml) was taken from each deer within 60 min after it was shot by making a small incision in the jugular vein using a scalpel [38]. To reduce the risk of contamination from the skin and hair, deer were either hung from a hoist or positioned with their head and neck down to collect the blood directly into a plastic cup. Once the blood was collected, it was immediately pipetted into EDTA anticoagulant tubes (3 × 10 ml) for trace element analysis and serum separator tubes (3 × 10 ml) for serum protein analysis. All tubes were brought to the slaughterhouse where the blood in the serum separator tubes was centrifuged for 10 min at 2561 × *g* after which the serum was pipetted into 5-ml cryotubes and kept cool at 4–6°C in a portable electric cooler box. Within 24 h, the cooled serum samples were delivered to the Veterinary Diagnostic Laboratory, University of Copenhagen, Denmark for serum protein analyses (described below).

Samples of liver (50g), kidney (50g), and hair (5g) were collected from each deer within 2–3 h after it was shot and transferred into separate Ziplock bags. Samples were taken from the right liver lobe and right kidney of each deer. We collected the entire kidney because element concentrations can vary substantially between the cortical part and medullary part of the kidney [39, 40]. We incorporated both sections in our samples by mixing them together following the freeze-drying and grinding process (described below). Hair was collected by manually pulling it from the right rump. All samples as well as the blood in EDTA anticoagulant tubes were kept cool in a portable electric cooler box (4–6°C) and transported to Aarhus University, Denmark, where they were frozen at −20°C (typically within 4–8 h after collection) until further processing.

The frozen liver and kidney samples were cut into slices with the visceral membrane removed and lyophilized using a SCANVAC CoolSafe freeze-drier at −50 °C for 24 h. All liver and kidney samples were weighted before and after freeze-drying to calculate moisture content. Freeze-dried liver and kidney samples were subsequently ground into a powder using a laboratory mill Retsch® Mixer MILL MM 400, at 30Hz for 120s per sample. Powderized liver and kidney material was transferred into 5-mL cryotubes for each sample. Hair samples were cleaned of any visible debris using a tweezer, rinsed twice in 96% ethanol and mQ H_2_O, transferred into clean paper bags, and dried at 35°C for 24 h. All treated samples (liver, kidney, hair) and the frozen whole blood were transported to Toulouse, France, for trace element analyses (described below).

Throughout the sample collection and treatment process, powder free nitrile gloves and sterile, stainless-steel material (e.g., knives, tweezers, scalpels, grinding balls) were used to reduce risk of sample (cross) contamination. New gloves were used between each sampling event.

### Trace Element Analyses

For the liver, kidney, and hair samples, approximately 150 mg (147.6–169.3 mg) of dried and homogenized material was placed in acid cleaned (2% HNO_3_) digitubes (SCP Sciences 010-500-263) together with 3 mL of ultrapure nitric acid (HNO_3_; ~67–69%, OptimaTM Grade, Fisher) closed with airtight caps, and digested overnight at 90°C. After being cooled down to room temperature the digestions were then diluted in two steps; first the digest was transferred to 50-mL Falcon tubes and diluted with mQ H_2_O (3:42 digest:mQ H_2_O) to create a 45-mL mother solution that was in kept dark conditions at ~4°C until further processing. Second, a 5-mL aliquot of the mother solution was taken and diluted to 10 mL with mQ H_2_O before being analyzed for selected trace elements (i.e., Cu, Zn, Se, As, Cd, Pb) using a Thermo Scientific® iCap TQ-ICP-MS at the Observatoirie Midi-Pyrénées, Toulouse, France.

Samples of whole blood were digested following a protocol adapted from Brumbaugh et al. [41], i.e., ~1.5 mL of thawed blood was placed in acid (2% HNO_3_) cleaned digitubes to which 2 mL of ultra-pure HNO_3_ (Fisher Chemical, nitric acid HNO_3_, Optima Grade, 67–69%) was added. After 1 h, the tubes were sealed with airtight caps and placed on a hotplate at 90°C for 45 min. Digestions were then cooled down to room temperature, the caps removed and after adding 0.4 mL of high-purity 30% (v/v) H_2_O_2_ they were resealed and returned to the hotplate for an additional 45 min. Finally, after cooling for at least 15 min, a mother solution (MS) was made by transferring the digest to a clean 50-mL Falcon tube and adding mQ H_2_O up to the 30-mL mark. Prior to the ICP-MS analysis, the samples were diluted in a second step by transferring a 4-mL aliquot of the MS to a clean 15-mL Falcon tube and then diluted by adding mQ H_2_O up to the 10 mL mark.

Quality assurance of the of digestion and analytical protocol was confirmed using blanks and certified reference materials (i.e., ERM DB 001, IAEA 336, DOLT-5, DORM-4, IAEA-A-13, ClinCheck Level 1) that were included with every ten samples (Table S1 in Supporting Information). All sample preparations were performed using acid cleaned labware and under clean laboratory conditions (Clean room class 10000, ISO 7) at the Clean Room platform of LEFE-INP, Toulouse, France.

After the TQ-ICP-MS analyses, trace element concentrations in liver and kidney (μg g^−1^ dry weight (d.w.)) were converted to μg g^−1^ wet weight (ww.) using the percent moisture in each sample. This was done to align the unit of concentration measurements across all tissue types.

### Serum Protein Analyses

The analyses of the total serum protein concentration (biuret reaction), albumin (bromcresol green reaction), and the acute phase reactant iron (ferrozine reaction) were conducted using an automated spectrophotometrical analyzer (Atellica®Solution; Siemens Healthineers, Germany). Analysis of the acute phase protein serum amyloid A (SAA) was done using an immunoturbidometric assay (SAA-LZ; Eiken Chemical; ADVIA 1800; Siemens Healthineers, Germany). This assay has previously been used in fallow deer and red deer [38]. Serum protein electrophoresis (SPE) was conducted using capillary electrophoresis (Minicap, Sebia Italia Srl, Italy) according to the manufacturer’s instructions. Fraction limits for five protein fractions (albumin, alpha 1, alpha 2, beta, and gamma globulins) were based on conventions for mammalian species and performed by the same operator for all samples. Absolute values were determined by multiplying the total protein by the percent of each fraction identified by SPE. The analytical machines and assays were all part of daily and weekly internal laboratory quality controls and quarterly external quality controls. Parameter values measured during the serum protein analyses are provided in Table S2 in Supporting Information.

### Statistical Analyses

Variation and (dis)similarities in trace element concentrations between tissues and deer species were visualized using boxplots and biplots following the multivariate ordination technique principal component analysis (PCA). Statistical differences in trace element concentrations between the study sites, sex, and age-classes were tested for each tissue and deer species separately using the non-parametric Kruskal–Wallis rank sum test or the Mann–Whitney *U* test as assumptions of normality and homogeneity of variance among groups were not always met. Correlations in trace element concentrations between and within tissue samples were quantified using the non-parametric Spearman correlation coefficient (*r*_*s*_).

Statistical differences in health indicators (i.e., BCS and serum protein parameters) between deer species, study sites, sex, and age-classes were also estimated separately using ANOVA (model residuals were normally distributed based on Shapiro–Wilk test).

To estimate direct linkages between the various essential and non-essential trace elements and body condition of deer, the data were analyzed using generalized linear mixed models (GLMM). Separate GLMMs were constructed for each tissues type and deer species. The response variable in the GLMMs was body condition score (BCS). The predictor variables included the concentrations of all six trace elements, which were scaled prior to analyses to facilitate comparison of model coefficients. The categorical variables study site and sex-class were also included as fixed effects to incorporate potential differences in element concentrations between groups. The continuous variable “days since the start of the sampling process” was also included as a fixed effect to account for possible changes in BCS of deer over time for reasons other than trace element status. Age-class was fitted as a random intercept to account for unbalanced data between groups. Once the full GLMM was constructed, we adopted a multi-model inference technique based on model averaging [42]. To this end, the set of candidate models consisted of all possible combinations of the trace element predictor variables, with the other fixed and random variables always included. Potential collinearity between explanatory variables was handled by removing candidate models with a *r*_*s*_ ≥0.7 among trace element variables. From the remaining models, model-averaged coefficients and unconditional SEs for covariates were calculated. All GLMMs were fitted using the maximum-likelihood estimation, which is necessary when comparing mixed-effects models or models with different fixed effects [43]. Finally, the relative variable importance [*w*_+_(*j*)] for each covariate was calculated by summing the Akaike’s weights (*w*) across all the models in the set where variable *j* occurred. The larger the w_+_(*j*) the more important variable *j* is in explaining variation of the response variable [42].

### Modeling Direct and Indirect Linkages Between Trace Element Status and Multiple Health Indicators

To jointly estimate direct and indirect linkages between trace element status in tissue samples and multiple deer health indicators (long- and short-term), we relied on structural equation modeling (SEM). SEM is based on path analysis [44], which is a statistical regression technique that estimates the impact of a set of predictor variables on multiple, possibly interrelated, response variables. Furthermore, SEM extends path analysis by including unmeasured variables termed “latent variables” [44, 45], which can be used to summarize multivariate data, such as “trace element status” or biological concepts like “health status”, that are not directly measurable themselves. SEM is particularly valuable to evaluate the relative importance of direct and indirect pathways influencing a system [46, 47], while also accounting for confounding variables that influence the response variable(s) but that are not of primary interest to the study objective [48].

For each tissue type and deer species, a separate SEM was constructed. Each SEM contained three latent variables. The first latent variable was “Elemental status”, which was described by the concentrations (μg g^−1^ w.w.) of all six trace elements measured in a specific tissue. The second latent variable was “Serum protein status” described by the combination of total protein (g L^−1^), albumin (g L^−1^), and serum amyloid A (SAA: mg L^−1^). We restricted “Serum protein status” to these three metrics as they are commonly used in rapid basic health assessments of domestic and wildlife species [30]. The third latent variable was termed “System factors”, which included the categorical variables study site and sex to account for their potential confounding effects on elemental status. Age-class and the continuous variable “days since the start of the sampling process” were not considered as their effects in this system were minimal (see Results section). Thus, the latent variable “System factors” was included as a predictor of “Elemental status”, which in turn was included as a predictor for “Serum protein status”. The measured variable BCS was fitted as a response variable of “Elemental status” and “Serum protein status”. This was done to assess both potential direct and indirect effect of “Elemental status” on the long-term health indicator BCS and short-term health indicator “Serum protein status”, the primary objective of these analyses.

All SEMs were fitted using the robust maximum-likelihood estimator and model fit was evaluated by a chi-square (*χ*^2^) test and the indices: root mean squared error of approximation (RMSEA) and comparative fit index (CFI) [49], which were corrected for small sample sizes using the Swain’s correction factor [50]. In the *χ*^2^ test, a *p* value >0.05 indicates acceptable model fit, while cut-off values of acceptable model fit for the indices are: RMSEA ≤0.1 and CFI ≥0.90 [49, 50]. To compare path strength and effect-sizes across deer species and tissue types, all continuous parameters were scaled prior to model fitting and standardized coefficients are reported for the results.

All statistical analyses and modeling for this study were conducted using the software package R, version 4.2.3 [51] and results were considered statistically significant at *p* values <0.05.

## Results

Complete datasets of trace element concentrations in the blood, liver, kidney, and hair samples, serum protein parameters, and BCS were obtained for 20 fallow deer (9 females and 11 males) and 21 red deer (9 females and 12 males) across the four different study sites in Denmark (Table S3 in Supporting Information). These included 14 juveniles (*n*= 2 fallow deer, *n*=12 red deer), 17 sub-adults (*n*= 12 fallow deer, *n*=5 red deer), and 10 adults (*n*= 6 fallow deer, *n*=4 red deer).

### Variation in Tissue-Specific Trace Element Profiles and Differences Between Study Sites, Sex-, and Age-Classes

**Fig. 2:**
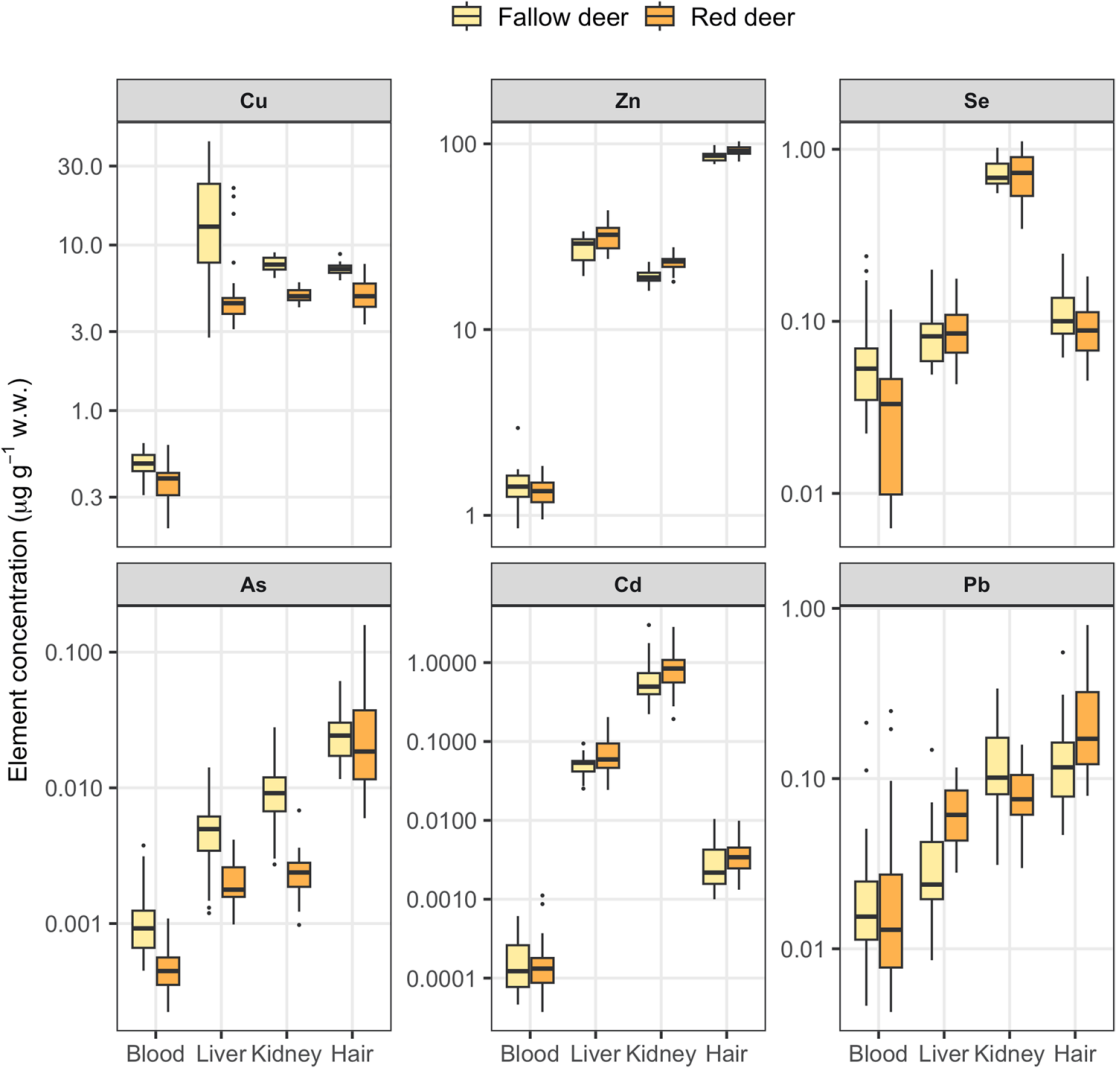
Boxplots showing the distribution of the essential (top row) and non-essential (bottom row) trace element concentrations (μg g^−1^ w.w.) measured in fallow deer (*Dama dama*, *N* = 20; yellow) and red deer (*Cervus elaphus*, *N*=21, orange) tissue samples collected during Nov–Dec 2021 at four sites in Denmark. The box shows the interquartile range of the data (25th to 75th percentile) with the median value indicated with a thick black horizontal line. The whiskers represent the range of the data within 1.5 times of the interquartile range while outliers are plotted as individual points. Note that the y-axis is on the logarithmic scale

Measured concentrations (μg g^−1^ wet weight (w.w.)) of essential (Cu, Zn, Se) and non-essential (As, Cd, Pb) trace elements in the blood, liver, kidney, and hair of fallow deer and red deer are presented in Fig. 2. For both deer species, concentrations of all six trace elements were lowest in blood as compared to the other tissues. Concentrations of Se and Cd were highest in the kidney, while Zn, As, and Pb concentrations were highest in hair (Fig. 2). Concentrations of Cu in fallow deer liver samples were two to four times higher than Cu in red deer liver (Fig. 2). The remaining five trace elements in liver of both deer species had intermediate concentrations when compared to other tissues (Fig. 2).

**Fig. 3:**
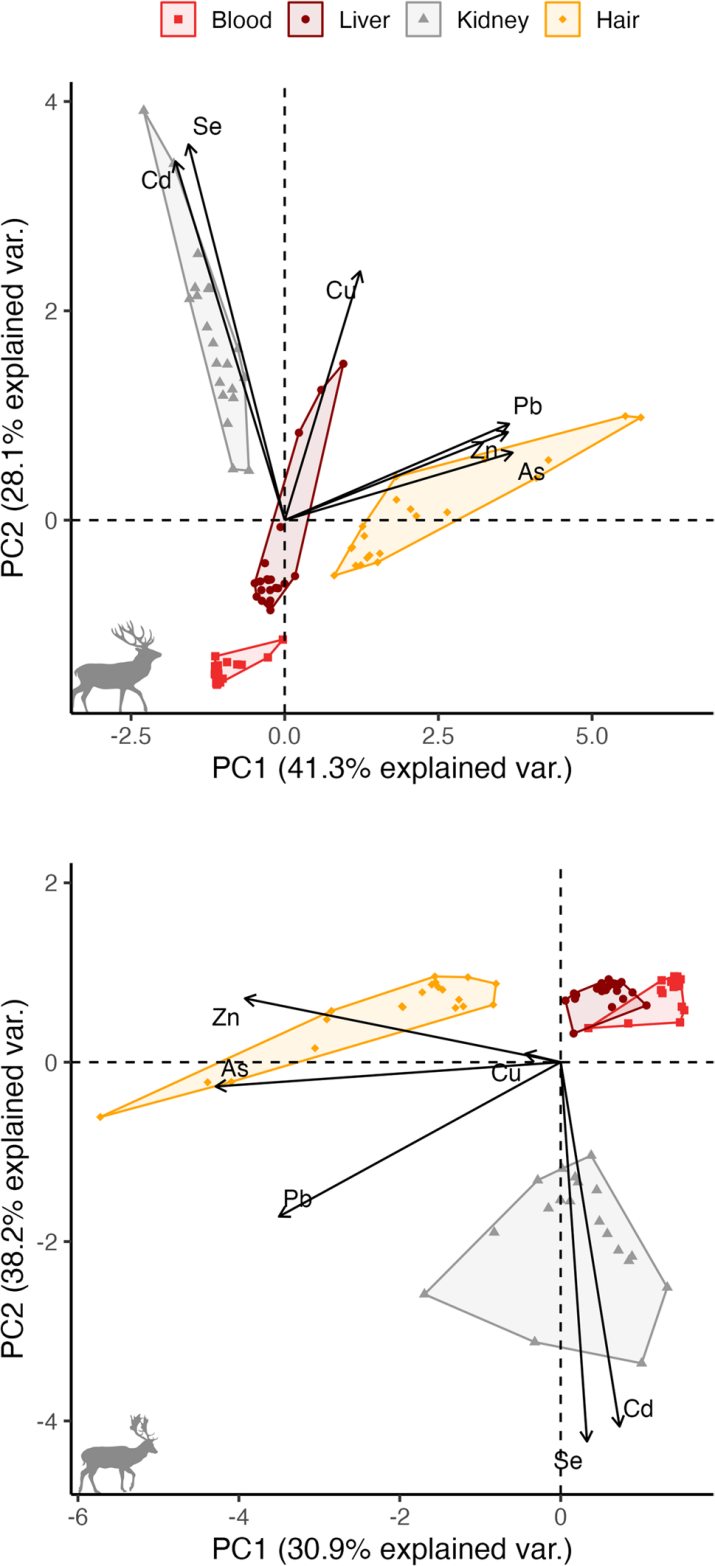
PCA ordination biplot showing the (dis)similarity in trace element (vectors) concentrations measured in different tissue types (blood: red squares, liver: brown circles, kidney: grey triangles, and hair: orange diamonds) collected from red deer (*Cervus elaphus*, *N*=21; top panel) and fallow deer (*Dama dama*, *N*=20; lower panel) at four sites in Denmark. The polygons were drawn to highlight overlap or separation in trace element profiles between tissues

The PCA ordination confirmed (dis)similarities in trace element concentrations between tissues, with the first two axes explaining 69.4% and 69.1% of the total variation in the data for red deer and fallow deer respectively (Fig. 3). In both deer species, elemental profiles in the different tissues were distinct without overlap in multi-element concentrations, except for blood and liver in fallow deer (Fig. 3).

In general, trace element concentrations varied most between study sites and sex, and least between age-classes for both deer species (see Fig. S1 for red deer and Fig S2 for fallow deer in the Supporting Information). Significant differences (*p*<0.05) in the average Se concentrations were detected between study areas and across all tissue types for both red deer and fallow deer. Moreover, Se concentrations were significantly higher in the blood, liver, and kidney of red deer males compared to females (Fig. S1 in the Supporting Information), while the reverse was detected in blood and liver for fallow deer (Fig. S2 in the Supporting Information). Concentrations of the non-essential trace elements As, Cd, and Pb were also significantly higher in the hair of males compared to the hair of females, a finding that was consistent for both deer species (Figs. S1 and S2 in the Supporting Information). Trace element concentrations were largely similar between age-classes in both deer species (Figs. S1 and S2 in the Supporting Information) with only a significant difference in trace element concentrations in blood (Zn higher in red deer juveniles than adults) and hair (As higher in fallow deer adults than juveniles).

### Correlations of Trace Element Concentrations Among and Within Tissues

Concentrations of Se were significantly (*p*<0.05) and positively correlated (*r*_*s*_>0.7) among all tissue types, a pattern that was evident for both deer species (Figs. S3 and S4 in the Supporting Information for red deer and fallow deer respectively). Concentrations of Cu in blood, liver and hair were also positively correlated (*p*<0.05) but only in red deer samples (Fig. S3 in the Supporting Information). For fallow deer, the only other statistically significant correlations detected were for concentrations of As between liver and kidney and for Cd between kidney and hair (Fig. S4 in the Supporting Information). No significant negative correlations were detected between elements in any of the tissues and deer species.

Within tissues, concentrations of the non-essential elements As, Cd, and Pb were significantly (*p*<0.05) and positively correlated in hair, a pattern that was evident in both deer species (Figs. S5 and S6 in the Supporting Information). For both deer species and within blood samples, concentrations of Cd and As were positively correlated, while Pb correlated positively with Cd in red deer blood and with Zn in fallow deer blood. No statistically significant (*p*<0.05) correlations were detected between element concentrations within kidney and liver samples for either deer species (Figs. S5 and S6 in the Supporting Information).

### Differences in Long- and Short-Term Health Indicators Between Deer Species, Study Sites, Sex-, and Age-Classes

**Fig. 4:**
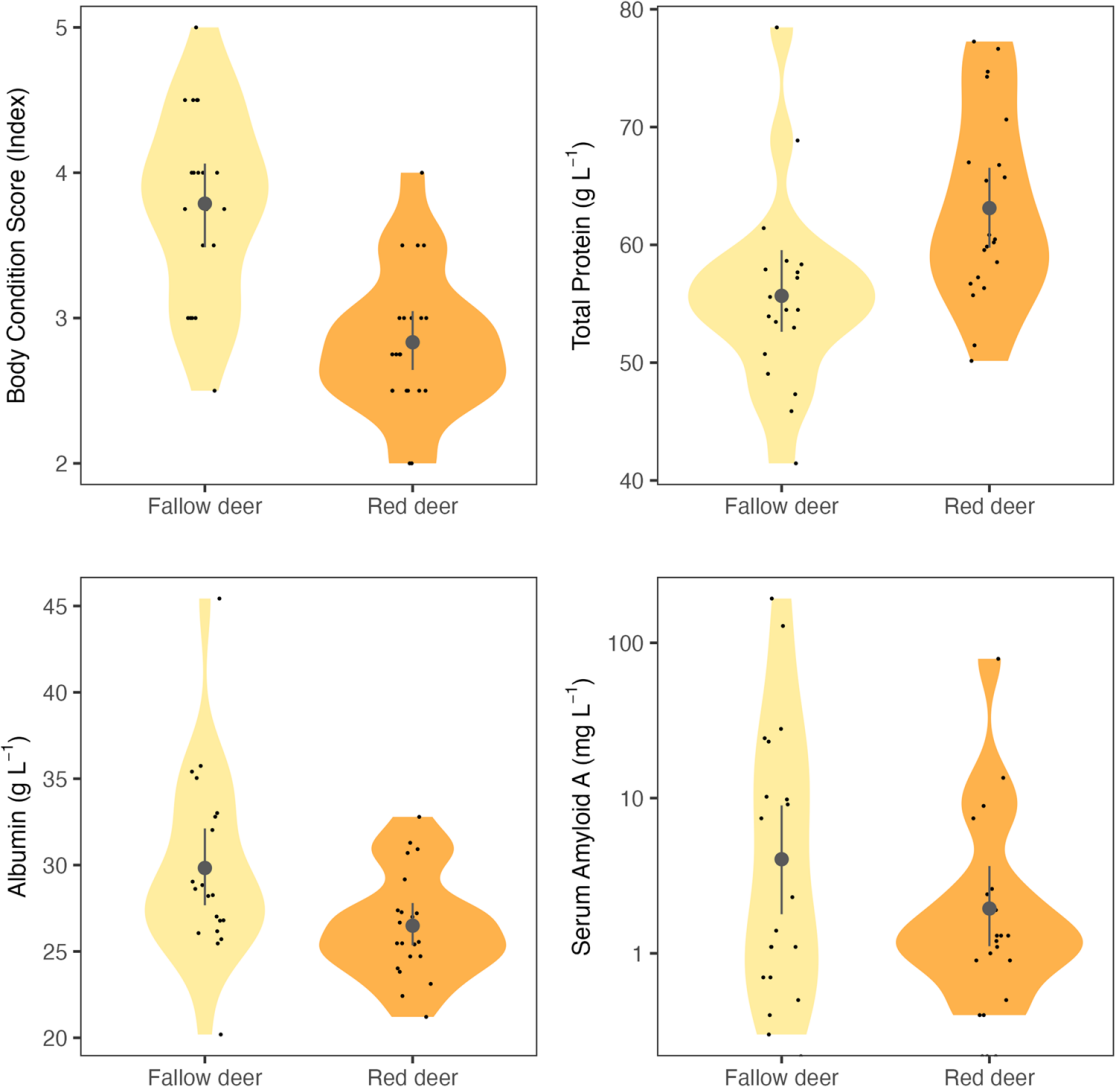
Violin plots showing the distribution of data points (small black dots) for the long-term health indicator Body Condition Score and three short-term health indicators (total protein, albumin, and serum amyloid A) measured in serum of fallow deer (*Dama dama*, *N* = 20; yellow) and red deer (*Cervus elaphus*, *N*=21, orange) collected during Nov–Dec 2021 at four sites in Denmark. The mean value and 95% confidence levels are provided for each health indicator and species by a grey circle with vertical lines

The long-term health indicator body condition score (BCS) differed between the two deer species (*p* <0.001) with a mean BCS of 2.83 (min=2, max=4) for red deer and a mean BCS of 3.79 (min=2.5, max=5) for fallow deer (Fig. 4). Moreover, the average BCS of red deer and fallow deer differed between study sites (*p* = 0.011 and *p* = 0.040 respectively), but not between sex- (*p* = 0.122 and *p* = 0.342) and age-classes (*p* = 0.287 and *p* = 0.077).

Out of the short-term health indicators considered here (Fig. 4), total protein (TP) and albumin differed between deer species (*p* =0.005 and *p*=0.018 respectively) while serum amyloid A (SAA) did not (*p*=0.165). Average TP, albumin, and SAA levels did not differ between sex- and age- classes for either deer species (*p*>0.05 for all tests). Serum protein parameters also did not differ between study sites for red deer (*p*>0.05 for all tests) but they did for fallow deer in the case of TP (*p* <0.001) and albumin (*p*=0.002).

### Direct Effects of Tissue-Specific Trace Element Concentrations on Body Condition Scores

**Fig. 5:**
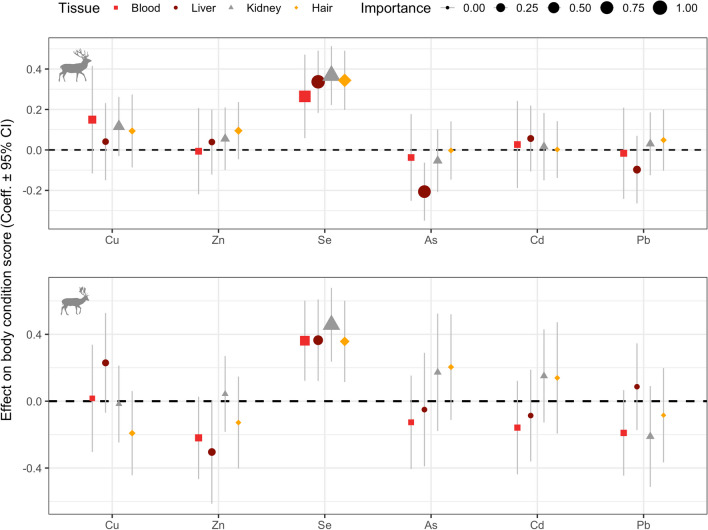
Model coefficients ± 95% confidence intervals of tissue-specific essential and non-essential trace element concentrations on body condition scores (BCS) of red deer (*Cervus elaphus*, *N*=21) in the top panel and fallow deer (*Dama dama*, *N*=20) in the lower panel. Size of the model coefficients varies according to variable importance (i.e., the larger the size the more important that element is in explaining variation in BCS). Confidence intervals that overlap with 0 (the horizontal dashed line) indicate a lack of a statistically significant (*p*<0.05) effect on BCS. Note that the effects of predictor variables study area, sex- and age-class as well as days since start of sampling are not plotted but were included in the models to account for possible changes in BCS of deer for reasons other than trace element status

For both deer species, Se concentrations had a consistently positive association with BCS and across all tissue types (Fig. 5). Moreover, the variable importance of Se was consistently high with *w*_+_(*j*) >0.5 for all tissues and both deer species (Fig. 5). For red deer, concentrations of As in the liver was an important parameter with *w*_+_(*j*) >0.75 and with a negative association to BCS. Yet, no direct linkages between concentrations of As and BCS were evident in any of the other tissues (Fig. 5). For the remaining trace elements, statistically significant associations with BCS were absent as the 95% confidence intervals (CI) overlapped with 0 (i.e., *p*>0.05) and variable importance was generally low (*w*_+_(*j*) <0.5; Fig. 5). For fallow deer, a negative association between Zn concentrations in the liver and BCS were detected, while no direct linkages between Zn and BCS were evident in any of the other tissues (Fig. 5). Concentrations of Cd and Pb were not associated with variation in BCS in any of the tissues or deer species (Fig. 5). The parameter “days since the start of the sampling process” was included as a fixed effect but had only little effect on BCS of either deer species (*p*>0.05 in most models), suggesting that the duration of the sampling period did not bias the above analyses.

### Direct and Indirect Linkages Between Trace Element Status and Multiple Health Indicators

**Fig. 6:**
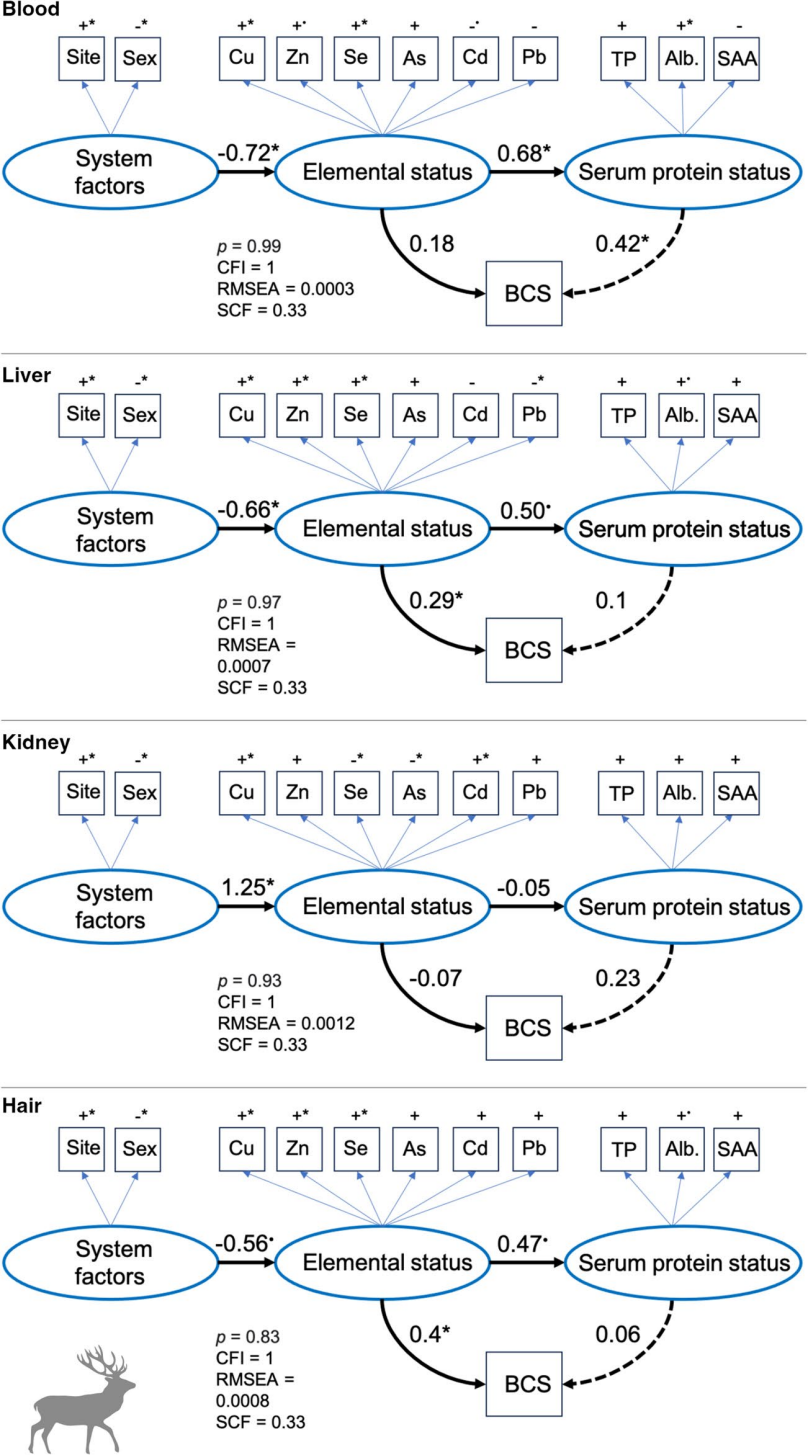
Path diagram showing linkages between trace element status of red deer (*Cervus elaphus*, *N*=21) in blood, liver, kidney, and hair to body condition score (BCS) and serum protein status (TP = total protein, Alb. = albumin, and SAA = serum amyloid A). Latent variables are drawn as ovals and measured variables are drawn as squares. Positive (+) and negative (−) symbols indicate the effect direction of that variable on the corresponding latent variable. Values indicate standardized path strength and significant pathways (*p*<0.05) are marked with an asterisk (*) while marginally significant pathways (*p*<0.1) are marked with a dot (^•^). Solid lines indicate direct effects, and the dashed line indicates an indirect effect of elemental status on BCS through serum protein status. Also provided are the *p* value for the chi-square (*χ*^2^) test and the model fit indices: root mean squared error of approximation (RMSEA) and comparative fit index (CFI) using the Swain’s correction factor (SCF). See Table S4 in Supporting Information for SEM outputs

For red deer, the output of the SEMs indicated that elemental status in blood had no direct, significant association with BCS. Instead, elemental status in blood had a significant association with serum protein status, with an indirect positive effect on BCS (Fig. 6). Elemental status measured in the liver and hair had direct positive linkages (*p*<0.05) with BCS but, in contrast to blood, no significant associations with serum protein status (*p*<0.1) or indirect linkages with BCS (Fig. 6). Elemental status in the kidney did not reveal any direct or indirect linkages to BCS and only a marginally significant (*p*<0.1) association between elemental status and serum protein status was detected (Fig. 6). See Table S4 in the Supporting Information for full SEM output and variance of measured and latent variables.

**Fig. 7:**
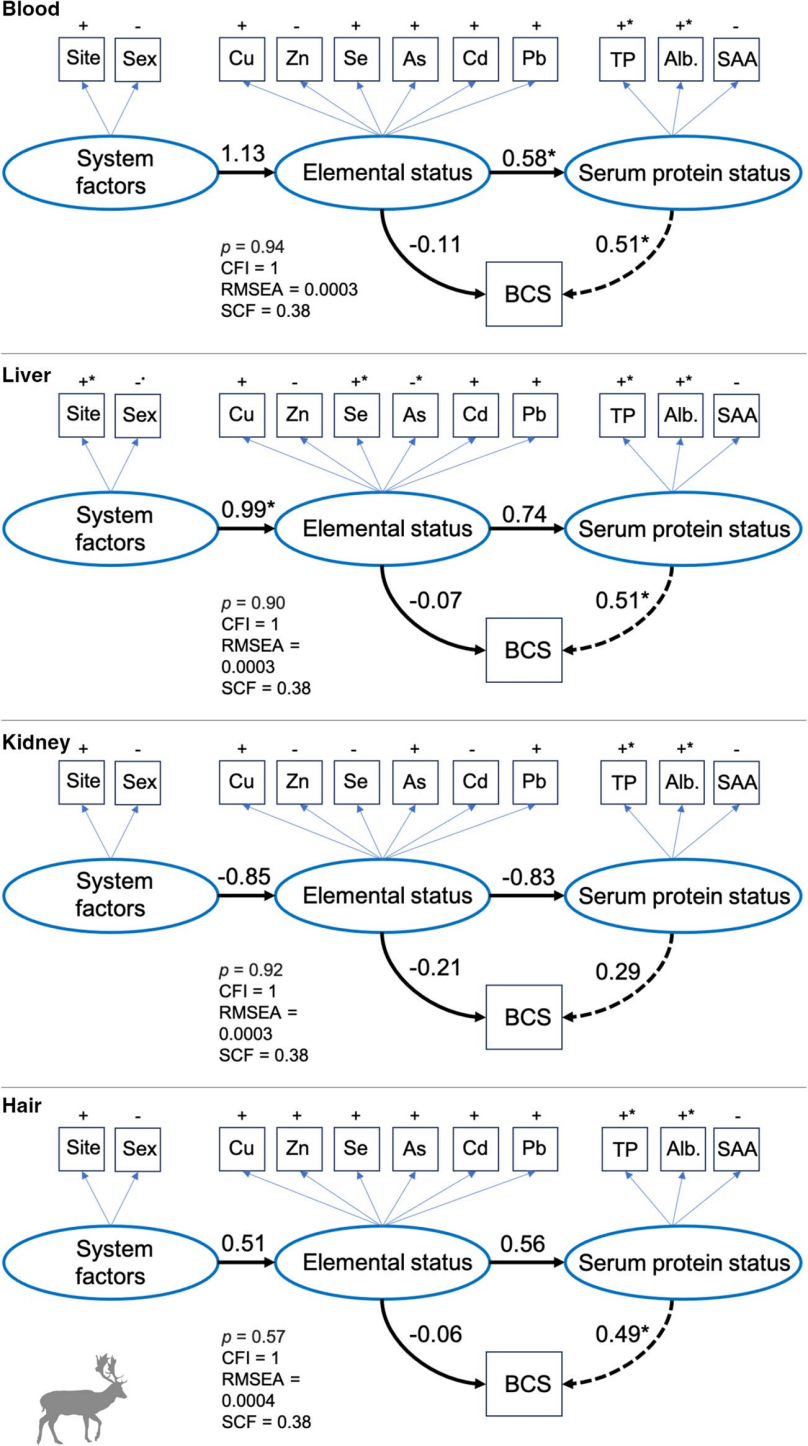
Path diagram showing linkages between trace element status of fallow deer (*Dama dama*, *N*=20) as determined in blood, liver, kidney, and hair to body condition score (BCS) and serum protein status (TP = total protein and Alb. = albumin, and SAA = serum amyloid A). Latent variables are drawn as ovals and measured variables are drawn as squares. Positive (+) and negative (−) symbols indicate the direction of that variable on the corresponding latent variable. Values indicate standardized path strength and significant pathways (*p*<0.05) are marked with an asterisk (*) while marginally significant pathways (*p*<0.1) are marked with a dot (^•^). Solid lines indicate direct effects, and the dashed line indicates an indirect effect of elemental status on BCS through serum protein status. Also provided are the *p*-value for the chi-square (*χ*^2^) test and the model fit indices: root mean squared error of approximation (RMSEA) and comparative fit index (CFI) using the Swain’s correction factor (SCF). See Table S5 in Supporting Information for SEM outputs

For fallow deer, the output of the SEMs did not reveal any significant direct associations between tissue-specific elemental status and BCS (Fig. 7). However, similar to the red deer result, elemental status in blood had a significant association with serum protein status, with an indirect positive effect on BCS (Fig. 7). For liver and hair, no significant associations were detected between elemental status and serum protein status (Fig. 7) although serum protein status had a significant positive association with BCS (Fig. 7). Elemental status in the kidney of fallow deer did not reveal any direct or indirect linkages to BCS and serum protein status. See Table S5 in the Supporting Information for full model output and variance of measured and latent variables.

Model fit was satisfactory for both deer species as all tissue-specific SEMs had *χ*^2^ test *p* values >0.05, RMSEA values ≤0.1 and CFI values ≥0.90 as determined through Swain’s correction factor (SCF) for small sample sizes (Figs. 6 and 7).

## Discussion

Monitoring trace element concentrations in animal tissue is an increasingly common practice in wildlife health assessments [12–14, 16]. However, tissue-specific pathways underlying element-health relationships are unknown for most species. Through complimentary statistical analyses, we show that the elemental status can vary substantially among tissues, with direct or indirect linkages to both long- and short-term health indicators of deer.

### Divergent Pathways Underlying Trace Element-Health Relationships

Using conventional multivariate regression, we found strong indications of a direct, positive relationship between Se and deer body condition, a pattern that was consistent across tissue types and deer species. This finding aligns with previous statements that Se is of critical importance in the nutritional and population ecology of deer and other free-ranging and domestic herbivores [52, 53]. Indeed, deficiency of Se has been shown to reduce individual growth, reproductive success, and overall population performance [54, 55]. Besides a negative association between As concentration in red deer liver and body condition, the multivariate regression did not reveal a statistical relationships between the remaining trace elements and deer body condition. These results may suggest that concentrations of Cu and Zn were within optimal ranges (i.e., homeostasis) and that concentrations of the non-essential elements Cd and Pb were below thresholds inducing negative health effects.

Se was the only essential trace element for which concentrations were significantly correlated among tissue types, indicating that the blood, liver, kidney, and hair may all be suitable tissues to monitor Se status in deer. None of the other trace elements, or combinations thereof, were consistently correlated within or across tissues (Figs. S3-S6 in the Supporting Information). This contrasts with findings of previous studies on cattle [56] and deer in other regions of Europe [57, 58] in which, for example, Cd and Zn were strongly and positively correlated in the kidney of sampled animals. Due to limited sample size, however, within and across-tissue element correlations in our study were estimated using all tissue-specific samples combined. As such, we did not explicitly test for possible age- and sex-effects on within and across tissue correlations or element interactions, which are likely to be important [58], and should be investigated in more detail within a wildlife health context.

Based on the SEM results, we uncovered clear tissue-specific and divergent pathways in trace element-health relationships, with notable differences between deer species. Discrepancies between model results emphasize the complexities underlying element-health relationships with important implications for the identification and effectiveness of tissue types to be used in wildlife health monitoring programs. SEMs are often used in long-term biological studies to investigate causes and consequences of environmental change [59], but their application to trace element data is rare [60]. Major advantages of SEM over conventional regression analyses are that variables can be used as response and explanatory variables at the same time, and that multiple parameters can be used to summarize a biological condition or state that is difficult to measure empirically [47]. In our case-study, concentrations of tissue-specific trace elements were used to describe a single latent variable i.e., trace element status, which, to some extent, incorporates dependencies among elements through potential antagonistic or synergetic effects [61]. Similarly, the latent variable serum protein status was summarized by total protein, albumin, and SAA, which are important parameters in the diagnosis of acute inflammation, infection, and malnutrition [30]. Through this model formulation, we found no direct linkages between elemental status in blood and the long-term health indicator body condition of both deer species. Instead, elemental status in blood was directly linked to serum protein status (short-term health) with an indirect positive association to deer body condition. It thus appears that blood is primarily suitable to study and monitor shifts in short-term health of deer, which was expected given the high turnover rate of element concentrations in this tissue.

Elemental profiles in liver and hair were directly linked to red deer body condition with weak associations to serum protein status. The similarity in results of liver and hair was expected and highlights that hair is a suitable biomarker to monitor element-induced shifts in long-term health, at least for red deer, as also proposed for other wildlife species [24–27]. The use of hair in element-health assessments has several benefits over traditionally used organ tissue as it can be repeatedly collected from the same individual using non-invasive methods and it can be stored at room temperature, which is ideal to assess nutritional or health status of protected or endangered species [26, 62].

Surprisingly, SEM results revealed no apparent association between elemental status in the storage organ kidney and the long- and short-term health indicators of both deer species. Previous studies have reported negative effects of presumed Cu deficiency and Cd toxicity based on kidney samples on the health of various ungulates [63, 64]. Note, however, that concentrations of the non-essential element Cd were 4 to 5 times higher than measured in kidney samples here [63]. Thus, possible explanations for our kidney findings may be that concentrations of the essential element Cu were above the deficiency threshold and, moreover, that non-essential elements As, Cd, and Pb measured in kidneys were below thresholds inducing negative health effects, as also inferred from the multivariate analyses. This is a probable explanation given that the kidney is one of the main routes of excretion of non-essential metals [65]. We surmise, therefore, that kidney tissue is of limited value in deer or wildlife health assessment programs that operate in low contamination areas.

No apparent association between trace element status and long- or short-term health indicators were detected in fallow deer tissue, which contrasted with the findings for red deer. Red- and fallow deer have overlapping diets and are both intermediate feeders, meaning they are able to switch between browse and grass dominated plant material [66]. We therefore expected the pathways underlying potential trace element-health relationships to be comparable between species. Yet, SEM output suggested that short-term health indicators (i.e., serum protein status) were more influential on body condition of fallow deer, cancelling out the direct association of trace element status identified through multivariate regression. We posit that differences in overall body condition between deer species may have contributed to these opposing results. Indeed, the average body condition score of fallow deer was 3.8 ±0.52, implying that most individuals were in good body condition [37]. For comparison, the average ± SD body condition score of red deer sampled here was 2.8 ±0.49, which was significantly lower than of fallow deer (*p*<0.001; Fig. 4) and suggests that most individual red deer were classified as being in poor to moderate body condition [37]. Thus, these findings may signal that direct linkages between elemental status and health indicators are only quantifiable below a certain overall body condition and that the essential element concentrations in fallow deer tissues were within required ranges (i.e., homeostasis) for optimal growth and condition. Unfortunately, and in contrast to livestock, few dedicated studies have been done to quantify optimal ranges (i.e., toxicity or deficiency thresholds) of trace element concentrations within different tissues of wildlife species [3]. As such, we were unable to verify if deer in poor body condition were deficient in essential elements. Bridging this data and knowledge gap is critical as it would greatly facilitate wildlife health assessments and the design of potential management incentives to offset adverse health effects following single or multi-element deficiency or toxicity.

### Caveats and Future Prospects

The analytical approach used in this study provided novel insights into tissue-dependent linkages between trace element concentrations and deer health. Yet, the results are based on a moderate sample size of 20 fallow deer and 21 red deer. While model performance was considered satisfactory for small sample sizes, data availability limited model construction, and prevented the consideration of additional drivers influencing deer health. For example, parasite burden [67], population density [68], and environmental change [69] are factors known to impact body condition of deer. Considering additional drivers, next to trace element concentrations, in future studies is critical to obtain a more holistic understanding of variation in long-term health of deer and wildlife in general. Moreover, we see great potential in the application of hormones such as cortisol and estrogen as these have proven useful in the determination of short-term animal health and may even interact with trace element status [70, 71]. Finally, our study focused on six trace elements known to play a key role in large herbivore health and population performance [25, 55]. Considering a wider range of trace and macro elements as well as their interactions (e.g., Cu and Mo absorption dynamics) may provide further insight into element-health relationships [5]. A valuable extension to this process would be to quantify and map spatial variation in the bioavailability of various trace elements in soils and the natural vegetation used by herbivorous species [55, 72]. While a monitoring program of heavy metal concentrations in arable soils of Denmark is currently in place [73, 74], similar data on concentrations of essential trace elements in soil and vegetation found in natural areas used by wildlife is currently scant. Filling this data gap is especially important for the management of deer populations or herds living within fenced areas as it allows for the evaluation of ongoing, or design potentially new, supplementary feeding programs. Future studies could take advantage of the SEM framework presented here to evaluate the impacts of all, or some of, the above parameters on changes in wildlife health. Ultimately, we stress that a variety of health or condition indicators should be used in biological and veterinary research as no “perfect” metric exists that can fully capture the multidimensionality of animal health and its linkages to trace elements.

## Conclusion

This study clearly shows that trace element status is tissue-specific and that no single sample matrix can be used to capture underlying long- and short-term health relationships in deer. To fully estimate trace element-health relationships the use of a variety of sample matrices is recommended. Nonetheless, liver and hair appeared suitable tissues to estimate direct and indirect linkages between trace element status and long- and short-term health indicators, at least when deer were in sub-optimal body condition, as for red deer in this study. Trace element status in blood appeared most suitable to monitor short-term health indicators, while kidney tissue appeared of little value in the estimation of element-health relationships in deer, at least based on our data from assumed non-contaminated areas. The quantification of (tissue-specific) deficiency and excess thresholds of essential and non-essential trace elements in free-ranging species is an important focus point for future research as these data will greatly facilitate wildlife monitoring programs and advance scientific understanding of direct and indirect linkages to health.

### Supplementary Information

ESM 1 (PDF 3626 kb)

## Data Availability

Data are available from the corresponding author upon a reasonable request.

## References

[1] Elser J (2006). Biological stoichiometry: a chemical bridge between ecosystem ecology and evolutionary biology. Am Nat.

[2] Trueman CN, Chung M-T, Shores D (2016). Ecogeochemistry potential in deep time biodiversity illustrated using a modern deep-water case study. Philos Trans R Soc Lond B Biol Sci.

[3] Mills CF, Bowie SHU, Webb JS (1997). Trace elements in animals. Philos Trans R Soc Lond B Biol Sci.

[4] Feinberg A, Stenke A, Peter T (2021). Reductions in the deposition of sulfur and selenium to agricultural soils pose risk of future nutrient deficiencies. Commun Earth Environ.

[5] Kaspari M, Powers JS (2016). Biogeochemistry and geographical ecology: embracing all twenty-five elements required to build organisms. Am Nat.

[6] Bhattacharya PT, Misra SR, Hussain M (2016). Nutritional aspects of essential trace elements in oral health and disease: an extensive review. Scientifica (Cairo).

[7] Kabata-Pendias A, Szteke B (2015). Trace elements in abiotic and biotic environments.

[8] Burger J (2008). Assessment and management of risk to wildlife from cadmium. Sci Total Environ.

[9] Massányi P, Massányi M, Madeddu R (2020). Effects of cadmium, lead, and mercury on the structure and function of reproductive organs. Toxics.

[10] Mandal P (2017). An insight of environmental contamination of arsenic on animal health. Emerg Contam.

[11] Gordon IJ, Hester AJ, Festa-Bianchet M (2004). The management of wild large herbivores to meet economic, conservation and environmental objectives. Journal of Applied Ecology.

[12] Cygan-Szczegielniak D, Stasiak K (2022). Effects of age and sex on the content of heavy metals in the hair, liver and the longissimus lumborum muscle of roe deer *Capreolus capreolus* L. Environ Sci Pollut Res Int.

[13] Srebočan E, Janicki Z, Crnić AP (2012). Cadmium, lead and mercury concentrations in selected red deer (*Cervus elaphus* L.) tissues from north-eastern Croatia. J Environ Sci Health A Tox Hazard Subst Environ Eng.

[14] Demesko J, Markowski J, Demesko E (2019). Ecotype variation in trace element content of hard tissues in the European roe deer (*Capreolus capreolus*). Arch Environ Contam Toxicol.

[15] Jarzyńska G, Falandysz J (2011). Selenium and 17 other largely essential and toxic metals in muscle and organ meats of red deer (*Cervus elaphus*) — consequences to human health. Environ Int.

[16] Klich D, Kitowski I, Łopucki R (2021). Essential differences in the mineral status of free-ranging European bison *Bison bonasus* populations in Poland: The effect of the anthroposphere and lithosphere. Sci Total Environ.

[17] Jutha N, Jardine C, Schwantje H (2022). Evaluating the use of hair as a non-invasive indicator of trace mineral status in woodland caribou (*Rangifer tarandus caribou*). PLOS ONE.

[18] Roug A, Swift PK, Gerstenberg G (2015). Comparison of trace mineral concentrations in tail hair, body hair, blood, and liver of mule deer (*Odocoileus hemionus*) in California. J Vet Diagn Invest.

[19] Blakley BR, Kutz SJ, Tedesco SC, Flood PF (2000). Trace mineral and vitamin concentrations in the liver and serum of wild muskoxen from Victoria Island. J Wildl Dis.

[20] Vermunt JJ, West DM (1994). Predicting copper status in beef cattle using serum copper concentrations. N Z Vet J.

[21] Squadrone S, Robetto S, Orusa R et al (2022) Wildlife hair as bioindicators of metal exposure. Biol Trace Elem Res. 10.1007/s12011-021-03074-6 10.1007/s12011-021-03074-6 35112231

[22] Cygan-Szczegielniak D, Stasiak K (2022). Concentration of selected essential and toxic trace elements in horse hair as an important tool for the monitoring of animal exposure and health. Animals.

[23] Oropesa A-L, Ramos A, Gómez L-J (2022). Toxic and essential metal levels in the hair of red deer (*Cervus elaphus*) and wild boar (*Sus scrofa*) for monitoring the contamination in protected areas of South-Western Spain. Environ Sci Pollut Res.

[24] Długaszek M, Kopczyński K (2014). Correlations between elements in the fur of wild animals. Bull Environ Contam Toxicol.

[25] Winter SN, Fernandez MD, Taylor KR, Wild MA (2022). Associations between hair trace mineral concentrations and the occurrence of treponeme-associated hoof disease in elk (<i>Cervus canadensis</>). BMC Vet Res.

[26] Rioux È, Pelletier F, Mosbacher JB (2022). Links between individual performance, trace elements and stable isotopes in an endangered caribou population. Glob Ecol Conserv.

[27] Mosbacher JB, Desforges J-P, Michelsen A (2022). Hair mineral levels as indicator of wildlife demographics?—a pilot study of muskoxen. Polar Res.

[28] Bremner I, Mills CF (1981). Absorption, transport and tissue storage of essential trace elements. Philos Trans R Soc Lond B Biol Sci.

[29] Kophamel S, Illing B, Ariel E (2022). Importance of health assessments for conservation in noncaptive wildlife. Conserv Biol.

[30] Cray C, Zaias J, Altman NH (2009). Acute phase response in animals: a review. Comp Med.

[31] Tothova C, Nagy O, Kovac G (2016). Serum proteins and their diagnostic utility in veterinary medicine: a review. Vet Med.

[32] Li A, Zhou Q, Mei Y et al (2022) The effect of urinary essential and non-essential elements on serum albumin: evidence from a community-based study of the elderly in Beijing. Front Nutr 9 10.3389/fnut.2022.946245 PMC9342688 35923200

[33] López-Alonso M (2012). Trace minerals and livestock: not too much not too little. Vet Sci.

[34] Cygan-Szczegielniak D (2021). The levels of mineral elements and toxic metals in the *Longissimus lumborum* muscle, hair and selected organs of red deer (*Cervus elaphus*) in Poland. Animals.

[35] Kruuk LEB, Slate J, Pemberton JM (2002). Antler size in red deer: heritability and selection but no evolution. Evolution.

[36] De Marinis AM, Chirichella R, Apollonio M, Hackländer K, Zachos FE (2020). Common fallow deer *Dama dama* (Linnaeus, 1758). Handbook of the mammals of Europe.

[37] Audige L, Wilson PR, Morris RS (1998). A body condition score system and its use for farmed red deer hinds. New Zealand J Agric Res.

[38] van Beest FM, Petersen HH, Krogh AKH (2023). Estimating parasite-condition relationships and potential health effects for fallow deer (*Dama dama*) and red deer (*Cervus elaphus*) in Denmark. Int J Parasitol Parasites Wildl.

[39] Wilk A, Wiszniewska B, Rzuchowska A (2019). Comparison of copper concentration between rejected renal grafts and cancerous kidneys. Biol Trace Elem Res.

[40] Svartengren M, Elinder CG, Friberg L, Lind B (1986). Distribution and concentration of cadmium in human kidney. Environ Res.

[41] Brumbaugh WG, Schmitt CJ, May TW (2005). Concentrations of cadmium, lead, and zinc in fish from mining-influenced waters of northeastern Oklahoma: sampling of blood, carcass, and liver for aquatic biomonitoring. Arch Environ Contam Toxicol.

[42] Burnham KP, Anderson DR (2002). Model selection and multimodel inference A practical information-theoretic approach.

[43] Pinheiro JC, Bates DM (2000). Mixed-effects models in S and S-Plus: statistics and computing.

[44] Shipley B (2016). Cause and correlation in biology: a user’s guide to path analysis, structural equations and causal inference with R.

[45] Grace JB, Bollen KA (2008). Representing general theoretical concepts in structural equation models: the role of composite variables. Environ Ecol Stat.

[46] Bollen KA (1987). Total, direct, and indirect effects in structural equation models. Sociol Methodol.

[47] Arif S, MacNeil MA (2023). Applying the structural causal model framework for observational causal inference in ecology. Ecol Monogr.

[48] Frauendorf M, Allen AM, Verhulst S (2021). Conceptualizing and quantifying body condition using structural equation modelling: a user guide. J Anim Ecol.

[49] Hu L, Bentler PM (1999). Cutoff criteria for fit indexes in covariance structure analysis: conventional criteria versus new alternatives. Struct Equ Modeling.

[50] Herzog W, Boomsma A (2009). Small-sample robust estimators of noncentrality-based and incremental model fit. Struct Equ Modeling.

[51] R Development Core Team (2023) R: a language and environment for statistical computing

[52] Flueck WT, Smith-Flueck JM, Mionczynski J, Mincher BJ (2012). The implications of selenium deficiency for wild herbivore conservation: a review. Eur J Wildl Res.

[53] Hall JA, Bobe G, Vorachek WR (2013). Effects of feeding selenium-enriched alfalfa hay on immunity and health of weaned beef calves. Biol Trace Elem Res.

[54] Flueck WT (1994). Effect of trace elements on population dynamics: selenium deficiency in free-ranging black-tailed deer. Ecology.

[55] van Beest FM, Schmidt NM, Stewart L (2023). Geochemical landscapes as drivers of wildlife reproductive success: insights from a high-Arctic ecosystem. Sci Total Environ.

[56] López Alonso M, Prieto Montaña F, Miranda M (2004). Interactions between toxic (As, Cd, Hg and Pb) and nutritional essential (Ca, Co, Cr, Cu, Fe, Mn, Mo, Ni, Se, Zn) elements in the tissues of cattle from NW Spain. Biometals.

[57] Durkalec M, Kolenda R, Owczarek T (2017). Expression of metallothionein in the liver and kidneys of the red deer (Cervus elaphus L.) from an industrial metal smelting area of Poland. Ecotoxicol Environ Safety.

[58] Garcia MH, Moreno DH, Rodríguez FS (2011). Sex- and age-dependent accumulation of heavy metals (Cd, Pb and Zn) in liver, kidney and muscle of roe deer (*Capreolus capreolus*) from NW Spain. J Environ Sci Health A.

[59] Buncher C, Succop P, Dietrich K (1991). Structural equation modeling in environmental risk assessment. Environ Health Perspect.

[60] Kebonye NM, Eze PN, Ahado SK, John K (2020). Structural equation modeling of the interactions between trace elements and soil organic matter in semiarid soils. Int J Environ Sci Technol.

[61] Obhodas J, Tucak-Zorić S, Kutle A, Valković V (2007). Indications for synergetic and antagonistic effects between trace elements in the environment to human health. Coll Antropol.

[62] Flueck WT (2020). Nutrition as an etiological factor causing diseases in endangered huemul deer. BMC Res Notes.

[63] Pollock B (2006). Trace elements status of white-tailed deer (*Odocoileus virginianus*) and moose (*Alces alces*) in Nova Scotia.

[64] Frank A (1998). “Mysterious” moose disease in Sweden. Similarities to copper deficiency and/or molybdenosis in cattle and sheep. Biochemical background of clinical signs and organ lesions. Sci Total Environ.

[65] Davenport A, Kimmel PL, Rosenberg ME (2015). Chapter 36 - trace elements in patients with chronic kidney disease. Chronic Renal Disease.

[66] Spitzer R, Felton A, Landman M (2020). Fifty years of European ungulate dietary studies: a synthesis. Oikos.

[67] Chrétien E, De Bonville J, Guitard J et al (2023) Few studies of wild animal performance account for parasite infections: a systematic review. J Anim Ecol. 10.1111/1365-2656.13864 10.1111/1365-2656.13864 36480357

[68] Potapov A, Merrill E, Lewis MA (2012). Wildlife disease elimination and density dependence. Proc Biol Sci.

[69] Acevedo-Whitehouse K, Duffus ALJ (2009). Effects of environmental change on wildlife health. Philos Trans R Soc Lond B Biol Sci.

[70] Montillo M, Caslini C, Peric T (2019). Analysis of 19 minerals and cortisol in red deer hair in two different areas of the Stelvio National Park: a preliminary study. Animals.

[71] Wojnarowski K, Podobiński P, Cholewińska P (2021). Impact of estrogens present in environment on health and welfare of animals. Animals.

[72] French AS, Shaw D, Gibb SW, Taggart MA (2017). Geochemical landscapes as drivers of trace and toxic element profiles in wild red deer (*Cervus elaphus*). Sci Total Environ.

[73] Jensen J, Larsen MM, Bak J (2016). National monitoring study in Denmark finds increased and critical levels of copper and zinc in arable soils fertilized with pig slurry. Environ Pollut.

[74] Bak J, Jensen J, Larsen MM (1997). A heavy metal monitoring-programme in Denmark. Sci Total Environ.

