# Supplementary material for: Direct and Indirect Linkages Between Trace Element Status and Health Indicators - a Multi-tissue Case-Study of Two Deer Species in Denmark
Source: Biol Trace Elem Res. 2023 Nov 2;202(8):3623–38. doi: 10.1007/s12011-023-03926-3 (PMC11144132; doi:10.1007/s12011-023-03926-3)
Supplement: Supplementary file 1 — (PDF 3626 kb) [file 12011_2023_3926_MOESM1_ESM.pdf]

SUPPORTING INFORMATION

(Tables and Figures)

For the article:

Direct and indirect linkages between trace element status and health indicators; a multi-tissue case-study of two deer species in Denmark

Floris M. van Beest[1,*] Niels M. Schmidt[1], Monica Lønborg Frederiksen[2], Anne K.H. Krogh[3,] Heidi H. Petersen[4], Sophia V. Hansson[5]

[1] Department of Ecoscience, Aarhus University, Frederiksborgvej 399, DK-4000 Roskilde, Denmark
[2] Vet Consult, Skovvej 58, 2920 Charlottenlund, Denmark
[3] Department of Veterinary Clinical Sciences, University of Copenhagen, Dyrlægevej 16, DK-1870, Frederiksberg, Denmark
[4] Center for Diagnostics, Technical University of Denmark, Kemitorvet, DK-2800 Kgs. Lyngby, Denmark.
[5] Laboratoire Ecologie Fonctionnelle et Environnement (UMR- 5245), CNRS, Université de Toulouse, Ave. de l'Agrobiopole, 31326, Castanet Tolosan, France
* Corresponding author: flbe@ecos.au.dk

Table S1. Results of quality control procedure (based on certified reference materials (CRM) of biotic tissues and detection limits (D.L. defined as 3x the standard deviation (SD) of blanks) of the iCap TQ-ICP-MS analyses used to measure concentrations of trace elements (Cu, Zn, As, Se, Cd, Pb) in tissue samples (blood, liver, kidney, hair) of fallow deer (*Dama dama*; N=20) and red deer (*Cervus elaphus*; N=21) collected during Nov-Dec 2021 in Denmark. See main article for detailed description of the digestion and analytical procedures. ND indicates no data.

	Cu	Zn	As	Se	Cd	Pb
D.L. (3 SD of blank)	0.000296	0.008916	0.000005	0.000039	0.000003	0.000020
**CRM ERM DB 001 Human hair (European Reference Materials)**						
This study[1] *(n = 5)*	34±2	195±6	0.039±0.002	2.9±0.03	0.129±0.010	2.00±0.14
Certified range	29-37	197-221	0.038-0.050	3.0-3.48	0.118-0.132	1.94-2.34
**CRM IAEA-336 Lichen (International Atmoic Energy Agency)**						
This study[1] *(n = 15)*	3.3±0.3	28.7±2.2	0.65±0.05	0.22±0.02	0.116±0.008	4.8±0.05
Certified range	3.1-4.1	27.0-33.8	0.55-0.71	0.18-0.26	0.100-0.134	4.3-5.5
**CRM DOLT-5 Dogfish liver (National research Council of Canada)**						
This study[1] *(n = 15)*	30.8 ±2.4	92.1±8.0	34.8±1.4	7.4±0.6	14.2±0.8	0.180±0.030
Certified range	32.6-37.4	99.9-110.7	32.2-37.0	6.5-10.1	13.9-15.1	0.130-0.194
**CRM DORM-4 Fish protein (National research Council of Canada)**						
This study[1] *(n = 10)*	14.60±2.18	46.4±2.6	6.42±0.39	3.45±0.17	0.295±0.013	0.257±0.036
Certified range	15.24-16.16	48.8-54.4	6.43-7.31	3.05-3.85	0.281-0.317	0.342-0.446
**CRM IAEA-A-13 Animal blood (International Atmoic Energy Agency)**						
This study[1] *(n = 9)*	3.3±0.05	12±1	ND	0.24±0.01	ND	0.16±0.002
Certified range	3.7-4.8	12-14	ND	0.15-0.31	ND	0.15-0.29
**CRM ClinCheck® Whole Blood Control Level I (Recipe Chemicals Germany)**						
This study[2] *(n = 3)*	0.559±0.075	3.30 ±0.44	0.00317±0.00022	0.0665±0.0033	0.00138±0.00007	0.0303±0.0052
Certified range	0.590-0.885	3.55-5.32	0.00242-0.00362	0.0665-0.0997	0.00118-0.00197	0.0301-0.0452

[1] *Ultrapure HNO_3 digestion using Digiprep tubes at 90°C overnight*

[2] *Ultrapure HNO_3 digestion using Digiprep tubes at 90°C for 45 min followed by addition of high-purity 30% (v/v) H_2O_2 at 90°C for 45 min.*

Table S2. Overview of parameter values of the serum protein analyses for red deer (*Cervus elaphus,* N=21) and fallow deer (*Dama dama,* N=20) sampled during Nov-Dec 2021 in Denmark.

Species	Parameter	Mean	StDev	Median	Min	Max	Range
Red deer	Albumin (g/L)	26.49	3.07	25.55	21.21	32.79	11.58
	Alpha1 (g/L)	2.78	1.02	3.02	0.06	4.25	4.19
	Alpha2 (g/L)	8.3	2.69	8.67	3.96	13.7	9.74
	Beta (g/L)	5.22	1.99	4.51	1.85	8.87	7.02
	Gamma (g/L)	20.33	6.02	18.6	13.65	35.13	21.48
	AG ratio	0.74	0.13	0.7	0.52	1	0.48
	Total protein (g/L)	63.12	8.01	60.47	50.15	77.26	27.11
	Serum Amyloid A (mg/L)	6	17.05	1.2	0	78.9	78.9
	Iron (µmol/L)	23.46	6.49	22.35	14	38	24
**Species**	**Parameter**	**Mean**	**StDev**	**Median**	**Min**	**Max**	**Range**
Fallow deer	Albumin (g/L)	29.83	5.39	28.44	20.19	45.43	25.24
	Alpha1 (g/L)	3.74	2.02	4.26	0.71	6.75	6.04
	Alpha2 (g/L)	7.43	1.78	7.26	4.38	11.03	6.65
	Beta (g/L)	4.58	1.82	4.3	1.62	7.73	6.11
	Gamma (g/L)	10.09	2.25	9.61	7.13	14.12	6.99
	AG ratio	1.17	0.24	1.14	0.85	1.63	0.78
	Total protein (g/L)	55.67	7.97	55.03	41.45	78.46	37.01
	Serum Amyloid A (mg/L)	22.13	49.27	1.85	0	192.8	192.8
	Iron (µmol/L)	26.36	6.96	24.1	17.5	45.7	28.2

Table S3. Overview of the number of fallow deer (*Dama dama*; N=20) and red deer (*Cervus elaphus*; N=21) sampled across species, sex, age-class and study site during Nov-Dec 2021 in Denmark.

Study site	Fallow deer					
	Female			Male		
	Juvenile	Sub-adult	Adult	Juvenile	Sub-adult	Adult
Dyrehaven	1	2	3	1	7	2
Gyldensteen Strand	0	2	1	0	1	0
Høstemark Skov	0	0	0	0	0	0
Tofte Skov	0	0	0	0	0	0
	Red deer					
Dyrehaven	0	0	0	6	2	2
Gyldensteen Strand	0	0	0	0	0	0
Høstemark Skov	2	1	0	0	0	0
Tofte Skov	3	1	2	1	1	0

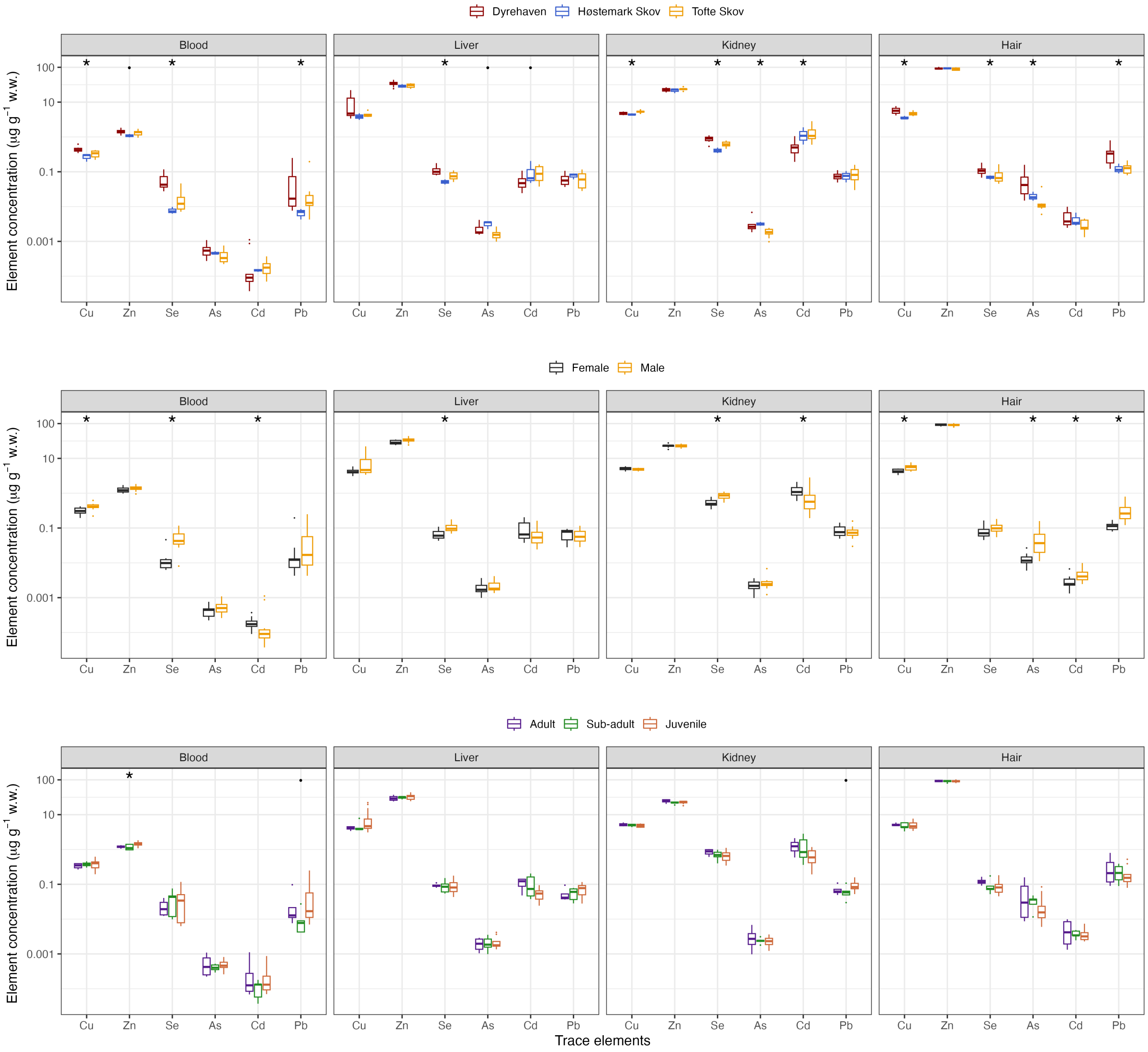

Figure S1. Boxplots showing the variation in trace element concentrations (µg g^{-1} w.w.) across study sites (top panel), sexes (middle panel), and age-classes (lower panel) measured in red deer (*Cervus elaphus*) tissue samples (N=21) collected during Nov-Dec 2021 in Denmark. Trace elements indicated with an asterisk (*) indicate a statistical difference ($p<0.05$) between groups while marginally significant differences ($p<0.1$) are marked with a dot (•) as determined with Kruskal-Wallis Rank Sum Test (3 group comparison) or the Mann-Whitney *U* test (2 group comparison). In all panels, the box shows the interquartile range of the data (25th to 75th percentile) with the median value indicated with a thick horizontal line. The whiskers represent the range of the data within 1.5 times of the interquartile range while outliers are plotted as individual points. Note that the y-axis is on the logarithmic scale.

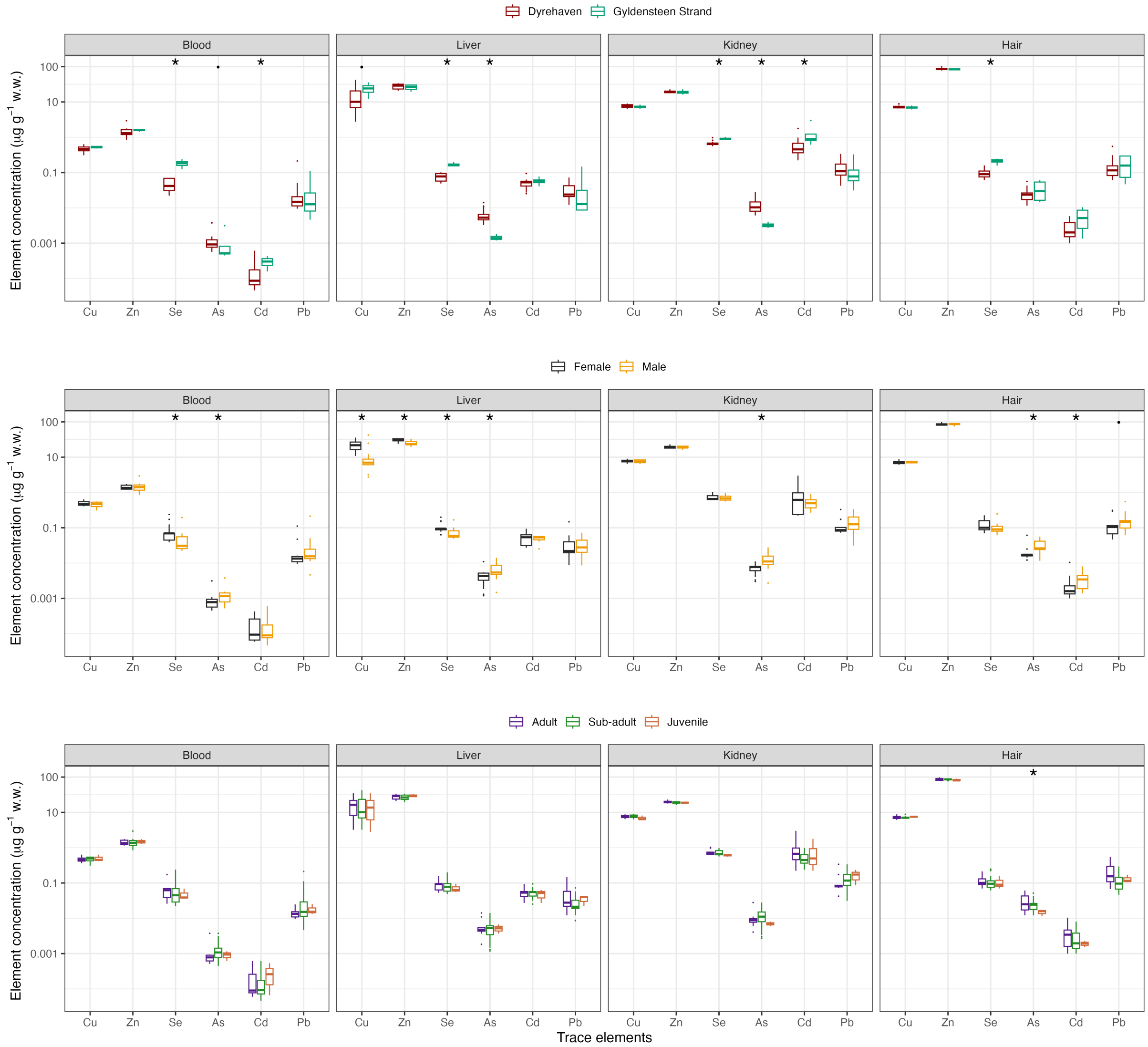

Figure S2. Boxplots showing the variation in trace element concentrations (μg g^{-1} w.w.) across study sites (top panel), sexes (middle panel), and age-classes (lower panel) measured in fallow deer (*Dama dama*) tissue samples (N=20) collected during Nov-Dec 2021 in Denmark. Trace elements indicated with an asterisk (*) indicate a statistical difference ($p<0.05$) between groups while marginally significant differences ($p<0.1$) are marked with a dot (•) as determined with Kruskal-Wallis Rank Sum Test (3 group comparison) or the Mann-Whitney *U* test (2 group comparison). In all panels, the box shows the interquartile range of the data (25th to 75th percentile) with the median value indicated with a thick horizontal line. The whiskers represent the range of the data within 1.5 times of the interquartile range while outliers are plotted as individual points. Note that the y-axis is on the logarithmic scale.

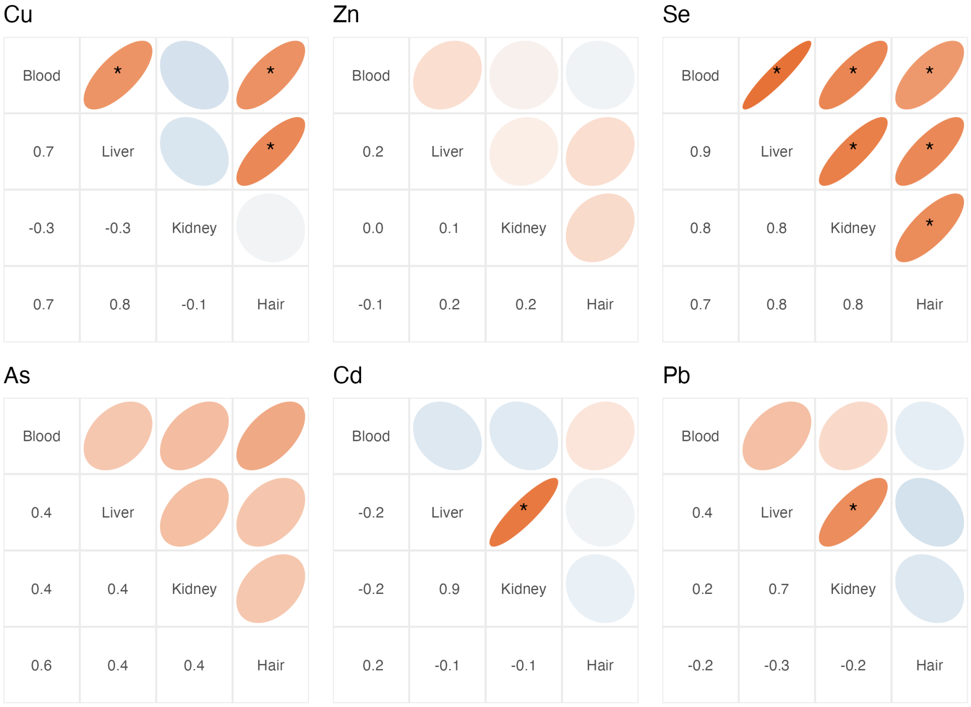

Figure S3. Correlation plots showing the direction (red = positive, blue = negative) and strength of the correlations (r_s values) in trace element concentrations among different tissue samples of red deer (*Cervus elaphus*, N=21) collected during Nov-Dec 2021 in Denmark. Tissue combinations marked with an asterisk (*) indicate a statistically significant correlation ($p<0.05$) for that element.

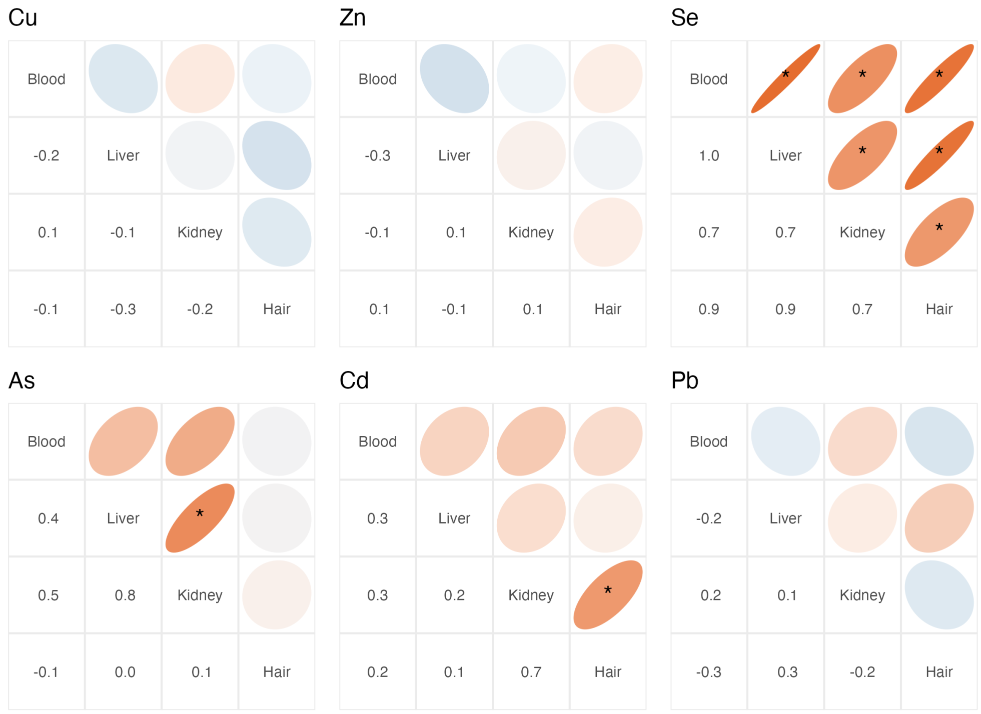

Figure S4. Correlation plots showing the direction (red = positive, blue = negative) and strength of the correlations (r_s values) in trace element concentrations among different tissue samples of fallow deer (*Dama dama*, N=20) collected during Nov-Dec 2021 in Denmark. Tissue combinations marked with an asterisk (*) indicate a statistically significant correlation ($p<0.05$) for that element.

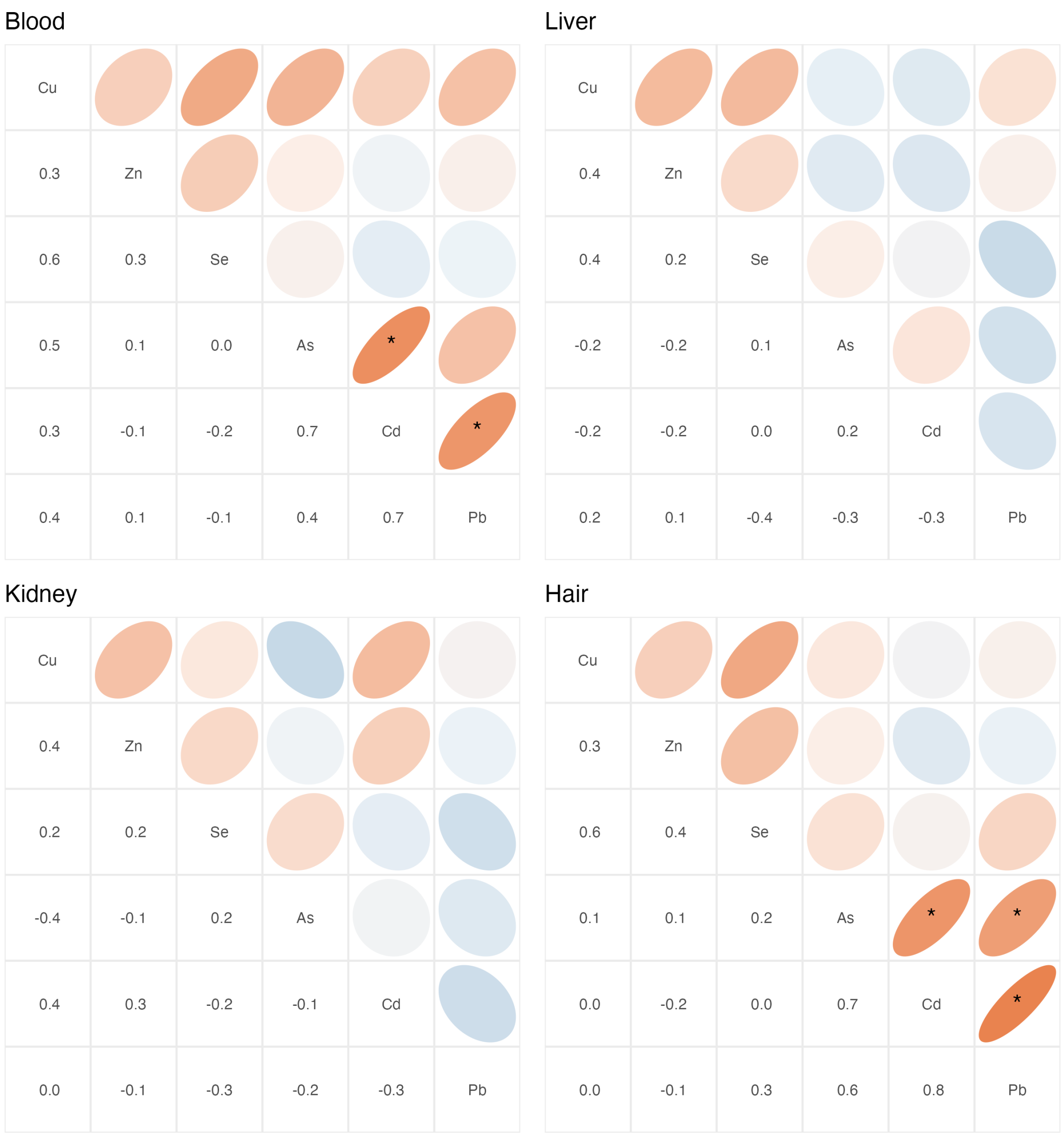

Figure S5. Plots showing the direction (red = positive, blue = negative) and strength of the correlations (r_s values) in trace element concentrations within tissue samples (N=21) of red deer (*Cervus elaphus*) collected during Nov-Dec 2021 in Denmark. Element combinations marked with an asterisk (*) indicate a statistically significant correlation (p<0.05) within that tissue.

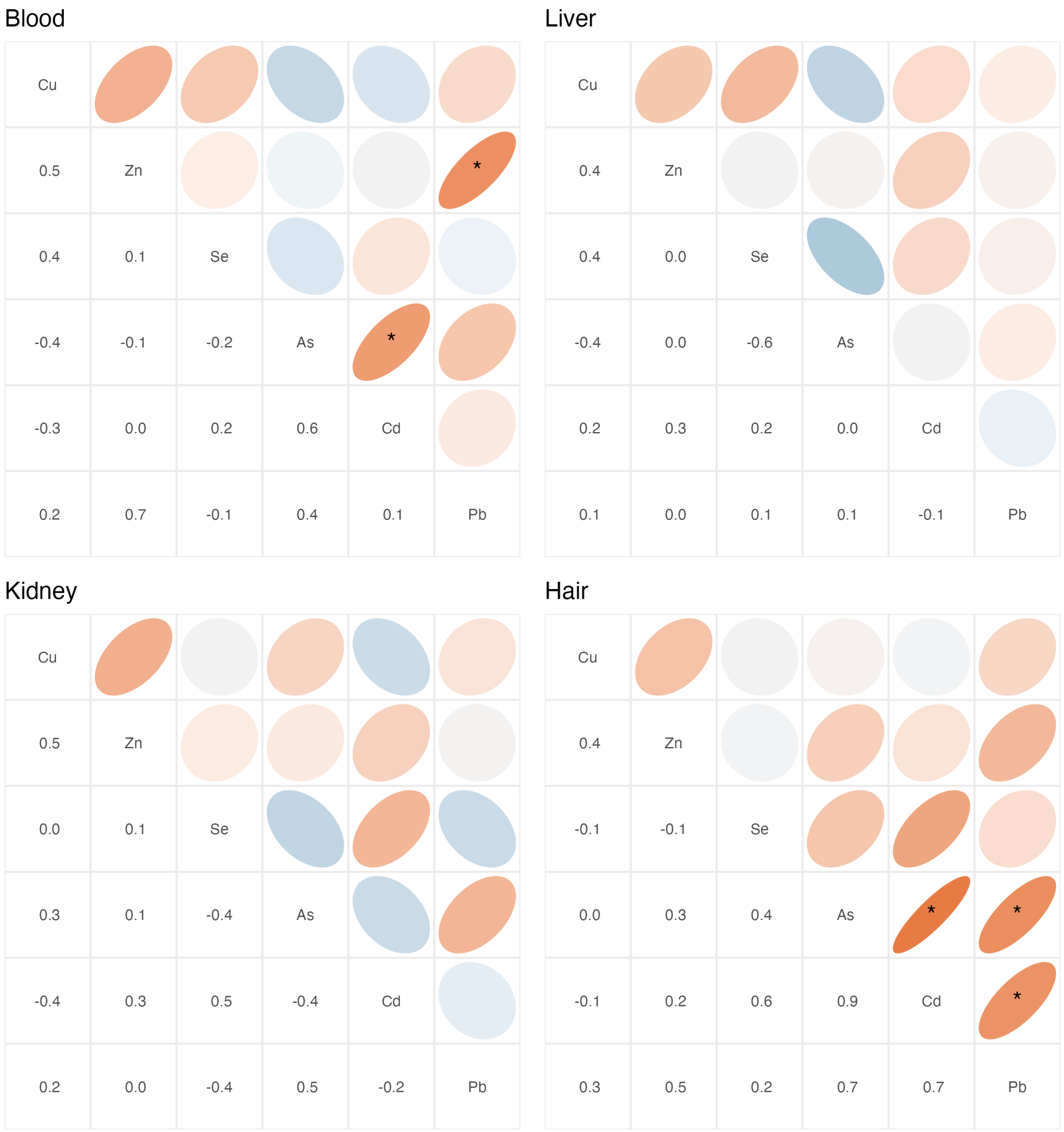

Figure S6. Plots showing the direction (red = positive, blue = negative) and strength of the correlations (r_s values) in trace element concentrations within tissue samples (N=20) of fallow deer (*Dama dama*) collected during Nov-Dec 2021 in Denmark. Element combinations marked with an asterisk (*) indicate a statistically significant correlation ($p<0.05$) within that tissue.

Table S4. Output of the structural equation models (SEMs) for red deer (*Cervus elaphus*, N=21) linking trace element status in different tissues (blood, liver, kidney, and hair) to multiple health indicators (BCS and Serum protein status). See main article for further details on SEM construction and meaning of variables. Model output forms the analytical basis for Fig. 6 in the main article.

Tissue	Variable	Estimate	Satandard error	*p*-value	Standardized path strength
**Blood**	**Latent Variables:**				
	System factors				
	Site	1.000	-	-	0.753
	Sex	-0.576	0.122	<0.001	-0.434
	Serum protein status				
	TP	1.000	-	-	0.522
	Alb.	1.722	0.853	0.044	0.898
	SAA	-0.600	0.598	0.316	-0.313
	Elemental status				
	Cu	1.000	-	-	0.537
	Zn	0.586	0.332	0.078	0.315
	Se	1.829	0.655	0.005	0.983
	As	0.068	0.236	0.773	0.037
	Cd	-0.337	0.187	0.071	-0.181
	Pb	-0.240	0.248	0.333	-0.129
	**Regressions:**				
	BCS ~ Elemental status	0.345	0.316	0.275	0.185
	BCS ~ Serum protein status	0.641	0.246	<0.001	0.428
	Elemental status ~ System factors	-0.516	0.223	0.020	-0.724
	Serum protein status ~ Elemental status	0.659	0.232	0.005	0.679
	**Variances**				
	.Site	0.281	0.137	0.041	0.281
	.Sex	0.057	0.049	0.249	0.057
	.TP	0.680	0.172	<0.001	0.680
	.Alb.	0.146	0.317	0.646	0.146
	.SAA	0.854	0.682	0.211	0.854
	.Cu	0.663	0.303	0.029	0.663
	.Zn	0.853	0.226	<0.001	0.853
	.Se	-0.014	0.131	0.913	-0.014
	.As	0.951	0.369	0.010	0.951
	.Cd	0.920	0.537	0.087	0.920
	.Pb	0.936	0.493	0.058	0.936
	.BCS	0.147	0.039	<0.001	0.147
	System factors	0.567	0.172	0.001	1.000
	.Serum protein status	0.147	0.112	0.189	0.539
	.Elemental status	0.138	0.053	0.010	0.477

**Liver**	**Latent Variables:**				
	System factors				
	Site	1.000	-	-	0.715
	Sex	-0.639	0.158	<0.001	-0.457
	Serum protein status				
	TP	1.000	-	-	0.474
	Alb.	2.037	1.155	0.078	0.965
	SAA	0.672	0.681	0.324	0.318
	Elemental status				
	Cu	1.000	-	-	0.484
	Zn	0.634	0.275	0.021	0.307
	Se	1.893	0.532	<0.001	0.916
	As	0.074	0.434	0.865	0.036
	Cd	-0.186	0.313	0.553	-0.090
	Pb	-0.676	0.305	0.026	-0.327
	**Regressions:**				
	BCS ~ Elemental status	0.604	0.214	0.005	0.292
	BCS ~ Serum protein status	0.214	0.211	0.311	0.101
	Elemental status ~ System factors	-0.452	0.182	0.013	-0.667
	Serum protein status ~ Elemental status	0.493	0.274	0.072	0.504
	**Variances**				
	.Site	0.337	0.129	0.009	0.337
	.Sex	0.036	0.057	0.528	0.036
	.TP	0.728	0.200	<0.001	0.728
	.Alb.	0.022	0.474	0.963	0.022
	.SAA	0.851	0.672	0.205	0.851
	.Cu	0.718	0.391	0.066	0.718
	.Zn	0.858	0.276	0.002	0.858
	.Se	0.114	0.144	0.428	0.114
	.As	0.951	0.293	0.001	0.951
	.Cd	0.944	0.315	0.003	0.944
	.Pb	0.845	0.182	<0.001	0.845
	.BCS	0.109	0.040	0.007	0.109
	System factors	0.511	0.173	0.003	1.000
	.Serum protein status	0.167	0.133	0.210	0.746
	.Elemental status	0.130	0.089	0.145	0.555
**Kidney**	**Latent Variables:**				
	System factors				
	Site	1.000	-	-	0.926
	Sex	-0.381	0.101	<0.001	-0.353
	Serum protein status				
	TP	1.000	-	-	0.455
	Alb.	2.216	1.396	0.113	1.009

	SAA	0.657	0.670	0.327	0.299
	Elemental status				
	Cu	1.000	-	-	0.368
	Zn	0.350	0.262	0.181	0.129
	Se	-1.207	0.520	0.020	-0.444
	As	-0.812	0.381	0.033	-0.299
	Cd	1.322	0.530	0.013	0.487
	Pb	0.640	0.482	0.184	0.236
	**Regressions:**				
	BCS ~ Elemental status	-0.192	0.119	0.108	-0.071
	BCS ~ Serum protein status	0.513	0.331	0.121	0.234
	Elemental status ~ System factors	0.499	0.187	0.008	1.254
	Serum protein status ~ Elemental status	-0.063	0.176	0.720	-0.051
	**Variances**				
	.Site	-0.009	0.083	0.916	-0.009
	.Sex	0.120	0.052	0.020	0.120
	.TP	0.745	0.232	0.001	0.745
	.Alb.	-0.066	0.611	0.914	-0.066
	.SAA	0.863	0.676	0.202	0.863
	.Cu	0.817	0.193	<0.001	0.817
	.Zn	0.936	0.279	0.001	0.936
	.Se	0.755	0.183	<0.001	0.755
	.As	0.863	0.538	0.109	0.863
	.Cd	0.715	0.305	0.019	0.715
	.Pb	0.897	0.266	0.001	0.897
	.BCS	0.173	0.040	<0.001	0.173
	System factors	0.857	0.148	<0.001	1.000
	.Serum protein status	0.207	0.161	0.198	0.997
	.Elemental status	-0.078	0.064	0.224	-0.573
**Hair**	**Latent Variables:**				
	System factors				
	Site	1.000	-	-	0.799
	Sex	-0.512	0.176	0.004	-0.409
	Serum protein status				
	TP	1.000	-	-	0.463
	Alb.	2.132	1.276	0.095	0.988
	SAA	0.667	0.678	0.326	0.309
	Elemental status				
	Cu	1.000	-	-	0.662
	Zn	0.640	0.284	0.024	0.424
	Se	1.300	0.349	<0.001	0.860
	As	0.402	0.336	0.232	0.266
	Cd	0.105	0.344	0.761	0.069

Pb	0.503	0.395	0.203	0.333
**Regressions:**				
BCS ~ Elemental status	0.605	0.184	0.001	0.400
BCS ~ Serum protein status	0.120	0.137	0.383	0.056
Elemental status ~ System factors	-0.462	0.277	0.096	-0.557
Serum protein status ~ Elemental status	0.327	0.191	0.087	0.468
**Variances**				
.Site	0.210	0.139	0.131	0.210
.Sex	0.078	0.070	0.267	0.078
.TP	0.738	0.225	0.001	0.738
.Alb.	-0.024	0.547	0.965	-0.024
.SAA	0.857	0.670	0.201	0.857
.Cu	0.514	0.154	0.001	0.514
.Zn	0.773	0.191	<0.001	0.773
.Se	0.212	0.148	0.151	0.212
.As	0.882	0.492	0.073	0.882
.Cd	0.948	0.332	0.004	0.948
.Pb	0.841	0.385	0.029	0.841
.BCS	0.050	0.029	0.083	0.050
System factors	0.638	0.214	0.003	1.000
.Serum protein status	0.168	0.141	0.234	0.781
.Elemental status	0.302	0.169	0.074	0.689

Table S5. Output of the structural equation models (SEMs) for fallow deer (*Dama dama*, N=20) linking trace element status in different tissues (blood, liver, kidney, and hair) to multiple health indicators (BCS and Serum protein status). See main article for further details on SEM construction and meaning of variables. Model output forms the analytical basis for Fig. 7 in the main article.

Tissue	Variable	Estimate	Standard error	*p*-value	Standardized path strength
**Blood**	**Latent Variables:**				
	System factors				
	Site	1.000	-	-	0.403
	Sex	-0.370	0.289	0.200	-0.149
	Serum protein status				
	TP	1.000	-	-	0.938
	Alb.	0.991	0.125	<0.001	0.930
	SAA	-0.099	0.140	0.480	-0.093
	Elemental status				
	Cu	1.000	-	-	0.085
	Zn	-0.505	1.711	0.768	-0.043
	Se	9.075	8.404	0.280	0.773
	As	1.562	3.660	0.669	0.133
	Cd	4.644	5.074	0.360	0.396
	Pb	0.842	2.233	0.706	0.072
	**Regressions:**				
	BCS ~ Elemental status	-1.330	1.864	0.476	-0.113
	BCS ~ Serum protein status	0.541	0.146	<0.001	0.508
	Elemental status ~ System factors	0.239	0.234	0.307	1.130
	Serum protein status ~ Elemental status	3.403	1.181	0.030	0.582
	**Variances**				
	.Site	-0.002	0.038	0.954	-0.002
	.Sex	0.225	0.034	<0.001	0.225
	.TP	0.070	0.059	0.233	0.070
	.Alb.	0.086	0.060	0.152	0.086
	.SAA	0.941	0.590	0.111	0.941
	.Cu	0.943	0.283	0.001	0.943
	.Zn	0.948	0.586	0.106	0.948
	.Se	0.352	0.140	0.012	0.352
	.As	0.932	0.492	0.058	0.932
	.Cd	0.793	0.365	0.030	0.793
	.Pb	0.945	0.672	0.160	0.945
	.BCS	0.198	0.045	<0.001	0.198
	System factors	0.162	0.078	0.037	1.000
	.Serum protein status	0.582	0.242	0.016	0.662
	.Elemental status	-0.002	0.005	0.667	-0.277

**Liver**	**Latent Variables:**				
	System factors				
	Site	1.000	-	-	0.600
	Sex	-0.514	0.287	0.073	-0.353
	Serum protein status				
	TP	1.000	-	-	0.937
	Alb.	0.935	0.095	<0.001	0.876
	SAA	-0.084	0.132	0.524	-0.079
	Elemental status				
	Cu	1.000	-	-	0.340
	Zn	-0.445	0.877	0.612	-0.151
	Se	2.625	1.258	0.037	0.892
	As	-1.705	0.871	0.049	-0.579
	Cd	0.588	0.589	0.318	0.200
	Pb	0.427	1.139	0.708	0.145
	**Regressions:**				
	BCS ~ Elemental status	-0.219	0.588	0.710	-0.074
	BCS ~ Serum protein status	0.549	0.210	0.009	0.514
	Elemental status ~ System factors	0.842	0.444	0.048	0.991
	Serum protein status ~ Elemental status	2.037	1.573	0.195	0.739
	**Variances**				
	.Site	0.043	0.026	0.093	0.270
	.Sex	0.217	0.038	<0.001	0.875
	.TP	0.072	0.050	0.148	0.072
	.Alb.	0.183	0.059	0.002	0.183
	.SAA	0.944	0.594	0.112	0.944
	.Cu	0.835	0.286	0.004	0.835
	.Zn	0.927	0.159	<0.001	0.927
	.Se	0.154	0.067	0.021	0.154
	.As	0.614	0.262	0.019	0.614
	.Cd	0.910	0.378	0.016	0.910
	.Pb	0.929	0.501	0.064	0.929
	.BCS	0.188	0.044	<0.001	0.188
	System factors	0.160	0.054	0.003	1.000
	.Serum protein status	0.399	0.159	0.012	0.454
	.Elemental status	0.002	0.008	0.800	0.018
**Kidney**	**Latent Variables:**				
	System factors				
	Site	1.000	-	-	0.380
	Sex	-0.416	0.344	0.226	-0.158
	Serum protein status				
	TP	1.000	-	-	0.913
	Alb.	0.986	0.125	<0.001	0.900

	SAA	-0.060	0.151	0.691	-0.055
	Elemental status				
	Cu	1.000	-	-	0.170
	Zn	-0.223	2.282	0.922	-0.038
	Se	-4.920	6.546	0.452	-0.838
	As	3.225	3.877	0.406	0.549
	Cd	-3.291	4.584	0.473	-0.560
	Pb	2.164	2.662	0.416	0.368
	**Regressions:**				
	BCS ~ Elemental status	-1.212	1.215	0.318	-0.206
	BCS ~ Serum protein status	0.323	0.268	0.227	0.295
	Elemental status ~ System factors	-0.382	0.479	0.425	-0.851
	Serum protein status ~ Elemental status	-4.437	5.352	0.407	-0.828
	**Variances**				
	.Site	0.016	0.066	0.810	0.016
	.Sex	0.223	0.037	<0.001	0.223
	.TP	0.117	0.071	0.100	0.117
	.Alb.	0.141	0.069	0.041	0.141
	.SAA	0.947	0.598	0.113	0.947
	.Cu	0.921	0.225	<0.001	0.921
	.Zn	0.949	0.325	0.004	0.949
	.Se	0.248	0.133	0.062	0.248
	.As	0.649	0.331	0.050	0.649
	.Cd	0.636	0.275	0.021	0.636
	.Pb	0.814	0.359	0.023	0.814
	.BCS	0.171	0.032	<0.001	0.171
	System factors	0.144	0.095	0.129	1.000
	.Serum protein status	0.263	0.143	0.067	0.315
	.Elemental status	0.008	0.026	0.756	0.275
**Hair**	**Latent Variables:**				
	System factors				
	Site	1.000	-	-	0.399
	Sex	-0.370	0.289	0.200	-0.300
	Serum protein status				
	TP	1.000	-	-	0.928
	Alb.	0.954	0.118	<0.001	0.885
	SAA	-0.074	0.142	0.602	-0.069
	Elemental status				
	Cu	1.000	-	-	0.229
	Zn	0.244	1.170	0.835	0.056
	Se	3.009	4.352	0.489	0.575
	As	4.458	6.021	0.459	0.851
	Cd	5.084	7.125	0.476	0.971

Pb	3.613	4.358	0.407	0.690
**Regressions:**				
BCS ~ Elemental status	-0.332	0.360	0.356	-0.063
BCS ~ Serum protein status	0.537	0.157	0.001	0.498
Elemental status ~ System factors	0.246	0.288	0.393	0.516
Serum protein status ~ Elemental status	2.733	3.896	0.483	0.562
**Variances**				
.Site	-0.002	0.038	0.958	-0.013
.Sex	0.225	0.034	<0.001	0.910
.TP	0.089	0.074	0.226	0.089
.Alb.	0.167	0.074	0.024	0.167
.SAA	0.945	0.596	0.113	0.945
.Cu	0.943	0.283	0.001	0.993
.Zn	0.914	0.294	0.002	0.914
.Se	0.620	0.284	0.029	0.620
.As	0.225	0.082	0.006	0.225
.Cd	0.008	0.046	0.866	0.008
.Pb	0.474	0.303	0.117	0.474
.BCS	0.185	0.042	<0.001	0.185
System factors	0.160	0.054	0.003	1.000
.Serum protein status	0.589	0.198	0.003	0.684
.Elemental status	0.027	0.071	0.708	0.734
